# Pro‐atherogenic actions of signal transducer and activator of transcription 1 serine 727 phosphorylation in LDL receptor deficient mice via modulation of plaque inflammation

**DOI:** 10.1096/fj.202100571RR

**Published:** 2021-09-27

**Authors:** Wijdan Al‐Ahmadi, Thomas S. Webberley, Alex Joseph, Ffion Harris, Yee‐Hung Chan, Reem Alotibi, Jessica O. Williams, Alaa Alahmadi, Thomas Decker, Timothy R. Hughes, Dipak P. Ramji

**Affiliations:** ^1^ Cardiff School of Biosciences Cardiff University Cardiff UK; ^2^ Department of Microbiology and Immunology Max F. Perutz Laboratories University of Vienna Vienna Austria; ^3^ Systems Immunity Research Institute School of Medicine Cardiff University Cardiff UK

**Keywords:** atherosclerosis, extracellular signal‐regulated kinase 1/2, inflammation, LDL receptor deficient mice, signal transducer and activator of transcription‐1

## Abstract

Atherosclerosis is a chronic inflammatory disorder of the vasculature regulated by cytokines. We have previously shown that extracellular signal‐regulated kinase‐1/2 (ERK1/2) plays an important role in serine 727 phosphorylation of signal transducer and activator of transcription‐1 (STAT1) transactivation domain, which is required for maximal interferon‐γ signaling, and the regulation of modified LDL uptake by macrophages in vitro. Unfortunately, the roles of ERK1/2 and STAT1 serine 727 phosphorylation in atherosclerosis are poorly understood and were investigated using ERK1 deficient mice (ERK2 knockout mice die *in utero*) and STAT1 knock‐in mice (serine 727 replaced by alanine; STAT1 S727A). Mouse Atherosclerosis RT² Profiler PCR Array analysis showed that ERK1 deficiency and STAT1 S727A modification produced significant changes in the expression of 18 and 49 genes, respectively, in bone marrow‐derived macrophages, with 17 common regulated genes that included those that play key roles in inflammation and cell migration. Indeed, ERK1 deficiency and STAT1 S727A modification attenuated chemokine‐driven migration of macrophages with the former also impacting proliferation and the latter phagocytosis. In LDL receptor deficient mice fed a high fat diet, both ERK1 deficiency and STAT1 S727A modification produced significant reduction in plaque lipid content, albeit at different time points. The STAT1 S727A modification additionally caused a significant reduction in plaque content of macrophages and CD3 T cells and diet‐induced cardiac hypertrophy index. In addition, there was a significant increase in plasma IL‐2 levels and a trend toward increase in plasma IL‐5 levels. These studies demonstrate important roles of STAT1 S727 phosphorylation in particular in the regulation of atherosclerosis‐associated macrophage processes in vitro together with plaque lipid content and inflammation in vivo, and support further assessment of its therapeutical potential.

**Abbreviations**
BMDM: bone marrow‐derived macrophages
DGLA: dihomo‐gamma‐linolenic acid
ERK1/2: extracellular signal‐regulated kinase 1/2
IFN‐γ: interferon‐γ
IL: interleukin
JAK: Janus kinase
LDLR^−/−^
: LDL receptor deficient mice
LDH: lactate dehydrogenase
MCP‐1: monocyte chemotactic protein‐1
STAT1: signal transducer and activator of transcription 1
TBHP: *tert*‐butyl hydroperoxide
TG: triacylglycerol
VSMC: vascular smooth muscle cells

## 1 INTRODUCTION

Atherosclerosis is an inflammatory disorder of the vasculature that is regulated by the action of cytokines.^1^, ^2^, ^3^, ^4^, ^5^ Among the different cytokines, interferon‐γ (IFN‐γ) is emerging as one of the master regulators of the disease.^1^, ^6^, ^7^ IFN‐γ modulates several key atherosclerosis‐associated processes, including activation of M1 macrophage polarization, foam cell formation, the inflammatory response, and smooth muscle cell proliferation.^6^, ^7^ About 25% of the macrophage transcriptome is regulated by IFN‐γ.^6^, ^7^ Deficiency of the cytokine or its cognate receptors in mouse model systems attenuates atherosclerosis development and produces a more stable plaque phenotype.^6^, ^7^ In contrast, atherosclerosis development is potentiated by injection of the cytokine in such models.^6^, ^7^ The pro‐atherogenic actions of interleukin (IL)‐12 and ‐18 have also been attributed to increased production of IFN‐γ and many lipid lowering drugs attenuate the action of this cytokine.^1^

The actions of IFN‐γ are mainly mediated via the Janus kinase (JAK)‐signal transducer and activator of transcription (STAT)‐1 pathway.^6^, ^7^ Binding of the cytokine to its cognate receptors results in JAKs‐mediated phosphorylation of STAT1 on tyrosine 701, which then triggers the dimerization of the transcription factor, its translocation to the nucleus, and activation of gene transcription by binding to its recognition sequences in the regulatory regions of target genes.^6^, ^7^ In addition to tyrosine 701, phosphorylation of serine 727 (S727) is required for maximal activity.^6^, ^7^ Indeed, knock‐in mice expressing a mutant STAT1 where S727 has been substituted to alanine (S727A) are resistant to lipopolysaccharide‐induced septic shock and have reduced IFN‐γ‐induced expression of several genes in macrophages.^8^ IFN‐γ also activates several other signaling pathways though systematic studies that have investigated the impact of these on STAT1 S727 phosphorylation and/or cellular changes mediated by this cytokine have been limited.^6^

We have previously investigated the molecular mechanisms underlying the actions of IFN‐γ on macrophages in relation to atherosclerosis with some focused on kinases that are known to phosphorylate STAT1 S727.^9^, ^10^, ^11^, ^12^ Use of pharmacological inhibitors and RNA interference assays demonstrated that extracellular signal‐regulated kinase (ERK)1/2 played an important role in the IFN‐γ‐mediated phosphorylation, and thereby activation, of STAT1 on S727, the expression of four key genes implicated in atherosclerosis [chemokines monocyte chemotactic protein‐1 (MCP‐1), macrophage inflammatory protein‐1β, IFN‐γ‐induced protein‐10, and intercellular adhesion molecule‐1] and the uptake of modified lipoproteins by human macrophages.^13^ Interestingly, upregulation of IFN/STAT1 pathways has been found to correlate with in vivo lipid content of human plaques determined by magnetic resonance imaging.^14^ In addition, data mining of atherosclerotic plaque transcriptomics predicted STAT1‐dependent gene signature as a potential diagnostic tool for the progression of this disease.^15^ Furthermore, STAT1 has been found to be S727 phosphorylated in murine and human atherosclerotic lesions and ERK is also activated in atherosclerotic lesions of mice and rabbits.^16^, ^17^ Despite such associations, the roles of ERK1/2 and STAT1 S727 phosphorylation in atherosclerosis development in mouse model systems have not been investigated. We have analyzed here the impact of ERK1 deficiency (ERK2 knockout mice die *in utero* ^18^) and STAT1 S727A modification on several macrophage processes relevant to atherosclerosis in vitro and on the development of the disease in LDL receptor deficient mice (LDLR^−/−^) fed a high fat diet in vivo. Our studies reveal an important role in particular for STAT1 S727 phosphorylation in atherosclerosis development via modulation of plaque inflammation.

## 2 MATERIALS AND METHODS

### 2.1 Animals and reagents

All chemicals were from Sigma‐Aldrich (Poole, UK) or Thermo Fisher Scientific (Loughborough, UK) unless otherwise stated. Breeding pairs of LDLR^−/−^ and ERK1 deficient mice (ERK1^−/−^) were from the Jackson Laboratories (LDLRtm1Her/J and Mapk3tm1Gela/J, respectively). The generation of STAT1 S727A knock‐in mice (referred to as STAT1 S727A mice hereafter) has been previously described.^8^ All these mice were in the C57BL/6 background (backcrossed >10 times) and expanded locally in the Joint Biological Services of Cardiff University. LDLR^−/−^ mice were bred with ERK1^−/−^ and STAT1 S727A mice to generate LDLR^−/−^/ERK1^−/−^ and LDLR^−/−^/STAT1 S727A mice along with LDLR^−/−^ single knockouts that served as appropriate controls. The genotypes of the mice were confirmed by PCR on genomic DNA extracted from tail tips. All of the mice were housed in a light and temperature‐controlled facility (12‐h light–dark cycle, 22°C) in a pathogen‐free environment.

Eight‐week‐old male ERK1^−/−^, STAT1 S727A, or C57BL/6J control mice were used for the isolation of bone marrow‐derived macrophages (BMDM). For studies on atherosclerosis development, 8‐week‐old LDLR^−/−^, LDLR^−/−^/ERK1^−/−^, or LDLR^−/−^/STAT1 S727A mice were fed a high fat diet [21% (w/w) pork lard and 0.15% (w/w) cholesterol] (Special Diet Services, Witham, UK) for 12 or 24 weeks. The mice were sacrificed at the end of the experimental period, under anesthesia, by cardiac puncture and death was confirmed by palpitation. Mouse and organ weights were determined using an electronic scale. All studies and protocols were approved by the Cardiff University Institutional Ethics Review Committee and the United Kingdom Home Office, and experiments were performed in accordance with the Guide for the Care and Use of Laboratory Animals (NIH Publication No. 85‐23, revised 1996; Experimental licence 30/3365).

### 2.2 Isolation of BMDM

Femurs and tibias were isolated from the mice and muscle and cartilage removed. Both ends of the bones were then cut and the entire marrow was flushed out using RPMI 1640 with a 25‐gauge needle. Following centrifugation at 250× *g* for 5 min, the pellet was re‐suspended in 1 ml of red blood cells lysis buffer (150 mM NH_4_Cl, 10 mM KHCO_3_, 0.1 mM Na_2_EDTA, pH 7.4), incubated for about 1 min, and followed by the addition of 9 ml of RPMI 1640 containing 2 mM L‐glutamine supplemented with 10% (v/v) heat‐inactivated fetal calf serum (HI‐FCS), 100 U/ml penicillin, and 100 μg/ml streptomycin. The cell suspension was then passed through a 40 μm cell strainer before centrifugation as above. The pellet was then washed three times with PBS and re‐suspended in 20 ml of differentiation medium [DMEM containing 10% (v/v) HI‐FCS and 100 U/ml penicillin, 100 μg/ml streptomycin and 20 ng/ml of macrophage colony stimulating factor] in a humidified incubator containing 5% (v/v) CO_2_. Differentiation was carried out for 6 days followed by washing with PBS and use of the cells for the isolation of RNA or various cell‐based assays in complete medium [DMEM containing 10% (v/v) HI‐FCS, 100 U/ml penicillin, and 100 μg/ml streptomycin].

### 2.3 Cell viability and proliferation assays

The cells were incubated in complete media for 24 h and the lactate dehydrogenase (LDH) content in cell supernatants was determined using the Pierce LDH cytotoxicity assay as recommended by the manufacturers (Thermo Fisher Scientific). The remaining cells were then stained for 5 min with 0.2% (w/v) crystal violet solution in 10% (v/v) ethanol. Following washing of the cells three times with PBS, the intracellular crystal violet was solubilized with 0.1 M NaH_2_P0_4_ in 50% (w/v) ethanol and the absorbance read at 570 nm using a spectrophotometer.

### 2.4 Reactive oxygen species (ROS) and phagocytosis

ROS levels were monitored using the DCFDA/H2DCFDA—Cellular ROS Detection Assay Kit from Abcam (ab113851; Cambridge, UK). Phagocytosis was monitored by the uptake of fluorescently labeled *Escherichia coli* strain K‐12 using a Vybrant^TM^ Phagocytosis Assay Kit.

### 2.5 Macrophage cholesterol homeostasis

The receptor‐mediated uptake of Dil‐oxidized LDL (oxLDL) was determined by flow cytometry as our previous studies.^13^, ^19^, ^20^, ^21^ At least 10 000 events were counted. Macropinocytosis was monitored using fluorescently labeled dye Lucifer yellow (LY) as previously described.^22^ For cholesterol efflux, macrophages were incubated for 24 h with acetylated LDL (AcLDL; 25 μg/ml) and [4‐^14^C]cholesterol (0.5 μCi/ml) in media containing 0.2% (v/v) fatty acid‐free BSA as our previous studies.^19^, ^21^, ^23^ The cells were then treated for a further 24 h with apolipoprotein A‐I (10 μg/ml). Cell supernatant was collected, and the remaining cells solubilized with 0.2 M NaOH. The radioactivity in cells and supernatant was determined using a liquid scintillation counter. Cholesterol efflux was calculated as a percentage of radioactivity in the supernatant compared to total radioactivity (cells and supernatant).

### 2.6 Macrophage migration

Migration of BMDM through an 8 μm pore size membrane was determined using a modified Boyden chamber. BMDM (0.25 × 10^6^ cells) in serum free DMEM media [containing 0.2% (w/v) fatty acid‐free BSA and 1% (v/v) penicillin/streptomycin] were placed on the top chamber and 0.5 ml of complete DMEM containing vehicle or the chemoattractant monocyte chemotactic protein‐1 (MCP‐1 from PeproTech, London, UK; 20 ng/ml) was added to the bottom chamber. The set up was incubated for 24 h at 37°C in a humidified incubator containing 5% (v/v) CO_2_. The media in the top chamber together with any non‐migrated cells were then gently removed. The membrane was then placed over 10 µl of Fluoroshield Mounting Medium with DAPI on a glass slide, covered with a coverslip and sealed. The sum of fluorescently stained, migrated cells from five different fields on each slide were then counted using an Olympus BX61 fluorescence microscope.

### 2.7 Gene expression analysis

Total RNA was isolated from BMDM using Ribosol^TM^ according to the manufacturer's instructions (Amresco LLC, Ohio, USA). The RNA (typically 1000 ng) was then reverse transcribed using Moloney Murine Leukemia Virus reverse transcriptase as recommended by the manufacturer (Promega, Southampton, UK). The cDNA (10 ng) was then subjected to quantitative polymerase chain reaction (qPCR) using Atherosclerosis RT^2^ Profiler PCR Arrays (Qiagen, Manchester, UK). The arrays contain primers for 84 atherosclerosis‐associated and five housekeeping genes together with three negative, three positive, and one genomic DNA contamination controls. The reaction used SYBR Green JumpStart^TM^ Taq Readymix^TM^ for qPCR with a two‐step amplification process (melting at 95°C for 15 s and simultaneous annealing/extension at 60°C for 60 s) for 45 cycles in LightCycler® 96 Real‐Time PCR System (Roche, Welwyn Garden City, UK). Gene transcript levels were calculated using the comparative Ct method and normalized to five housekeeping genes [*β‐actin*; β‐2‐Microglobulin (*B2m*); glyceraldehyde 3‐phosphate dehydrogenase (*Gapdh)*; β‐Glucuronidase (*Gusb*); and heat shock protein HSP 90‐β (*Hsp90ab1*)*]* whose expression was found to be stable. For standard, RT‐qPCR, total RNA from BMDM was isolated as above, reverse transcribed into cDNA and then subjected to qPCR as our previous studies.^3^, ^4^, ^20^, ^21^ The sequences of the primers were 5′‐GCTCAGCCAGATGCAGTTAACG‐3′ and 5′‐GCTTGGTGACAAAAACTACAGCTTC‐3′ for *MCP‐1*; 5′‐TGGAAAACAGTTAATGACCAGCCA‐3′ and 5′‐TCCAGTAACAGCTGACATGTTTGT‐3′ for *ABCA1*; and 5′‐ACACCCGCCACCAGTTCGCCAT‐3′ and 5′‐CACACCCTGGTGCCTAGGGCGGCCCACGATG‐3′ for *β‐actin*.

### 2.8 Atherosclerotic lesion analysis

The hearts were perfusion fixed with PBS followed by 10% (w/v) formalin buffered saline. They were then frozen in optimum cutting temperature (OCT) embedding matrix. Serial sections of 7 μm were then cut starting from the three valve cusps of the aortic root,^24^ collected onto poly‐L‐lysine coated slides (VWR, Lutterworth, UK) and air‐dried for 1 h before storage at −80°C. Plaque size and lipid content were then determined using hematoxylin and Oil red O staining.^25^, ^26^ Plaque occlusion (i.e., percentage plaque of lumen) was calculated as (total area of plaque/total area of lumen) × 100. For collagen staining, the slides were first fixed in ice cold 4% paraformaldehyde for 5 min, washed with water for 5 min, stained with hematoxylin for 3 min, cleared under running water and stained for 5 min with Van Gieson's stain (Abcam; ab150667). The slides were then dehydrated with 100% ethanol for 5 min, cleared in 100% xylene for 15 min, mounted in DPX, sealed with a coverslip and imaged using Leica DMRB microscope (5× magnification, 2.5 objective).

Immunofluorescent staining was performed using the following molecule‐specific and appropriate isotype‐control antibodies as negative control (all from Abcam, Cambridge, UK): anti‐CD3 (ab5690; 1:100 dilution) for T cells; anti‐MOMA‐2 (ab33451; 1:100 dilution) for macrophages; and anti‐α‐smooth muscle cell actin (ab5694; 1:100 dilution) for smooth muscle cells. The slides were left to thaw and air dried at room temperature for 10 min and then immersed in ice‐cold acetone for 10 min to fix the aortic root cross‐section. The slides were then washed twice in PBS and non‐specific interactions blocked in blocking buffer [5% (v/v) donkey or chicken serum in 5% (w/v) BSA in PBS] for 30 min at room temperature. The slides were then incubated overnight at 4°C with the primary antibody or appropriate isotype controls. After washing twice with PBS, the slides were incubated with the secondary antibodies [Donkey anti‐rabbit AF488 or chicken anti‐rat AF594; both 1:500 dilution] for 1 h in the dark at room temperature. The slides were then incubated in 0.3% (w/v) Sudan Black B in 70% (v/v) ethanol for 20 min in the dark to prevent autofluorescence. After washing three times with PBS, the slides were mounted with DAPI‐mounting media (Vector Laboratories, Peterborough, UK) and imaged using epifluorescence microscopy. All subsequent analysis was carried out in a blinded fashion where possible using the Image J software.

### 2.9 Plasma lipid analysis

Blood was collected from the animals by cardiac puncture into heparin coated tubes. The plasma was then obtained by centrifugation (12 000× *g*) for 5 min. The plasma levels of total cholesterol, LDL/VLDL, and HDL were determined using the Cholesterol Assay Kit ‐HDL (ab65390) according to the manufacturer's instructions (Abcam, Cambridge, UK). The plasma levels of triacylglycerol (TG) were determined by the Clinical Biochemistry Laboratories, University Hospital Cardiff on an Aeroset automated analyzer (Abbott Diagnostics, Berkshire, UK).

### 2.10 Plasma cytokine levels

Plasma cytokine levels were determined using a V‐PLEX Plus Pro‐inflammatory Panel 1 Mouse Kit (Meso Scale Discovery, Maryland, USA) by the Central Biotechnology Services in the School of Medicine at Cardiff University.

### 2.11 Western blot analysis

An appropriate amount of RIPA buffer (typically 1‐2 ml) was added to 50 mg of liver and homogenized using a pestle and mortar. The homogenized tissue was then placed into a clean tube and vortexed thoroughly for 30 s followed by centrifugation at 2000× *g* for 3 min at 4°C. The supernatant was then transferred into a clean tube. Equal amounts of protein were then size fractionated by SDS PAGE and subjected to Western blot analysis as previously described^3^, ^4^, ^20^, ^21^ using antibodies that recognize ERK1/2 (Cell Signaling Technology, 9102; 1:250 dilution), phospho‐STAT1 S727 (Cell Signaling Technology, 9177; 1:250 dilution), or the housekeeping protein GAPDH (Santa Cruz Biotechnology; 1:5000 dilution).

### 2.12 Data analysis

Data are presented as the mean ± SEM and were tested for normality using the Shapiro–Wilk test. Single comparisons were carried out using the Student's *t* test (two‐tailed, unpaired). For multiple comparisons, one‐way ANOVA was employed with Tukey's post‐hoc analysis (equal variances) unless otherwise stated. When more than one factor was compared, a two‐way ANOVA was used with Sidak's post hoc test. Data values outside two standard deviations of the mean were classed as outliers and removed before statistical analysis. The results were regarded as significant when *p ≤ .05.

## 3 RESULTS

### 3.1 The effect of deficiency of ERK1 and STAT1 S727 phosphorylation on the macrophage expression of key atherosclerosis‐associated genes

The ERK1^−/−^ mice were obtained from the Jackson Laboratory and the generation of STAT1 S727A mice has been previously described.^8^ The absence of ERK1 or STAT1 serine 727 phosphorylated protein was first confirmed by Western blot analysis (Figure S1A). The effect of the genetic modifications on the expression of key atherosclerosis‐associated genes in BMDMs from control C57BL/6J animals or those containing the genetic changes were investigated using Mouse Atherosclerosis RT² Profiler PCR Arrays. The arrays allow analysis of the expression of 84 key genes implicated in atherosclerosis such as those involved in transcriptional regulation, lipid transport and metabolism, cell growth and proliferation, apoptosis, stress responses, blood coagulation and circulation, cell adhesion, and plaque stability.

**FIGURE 1:**
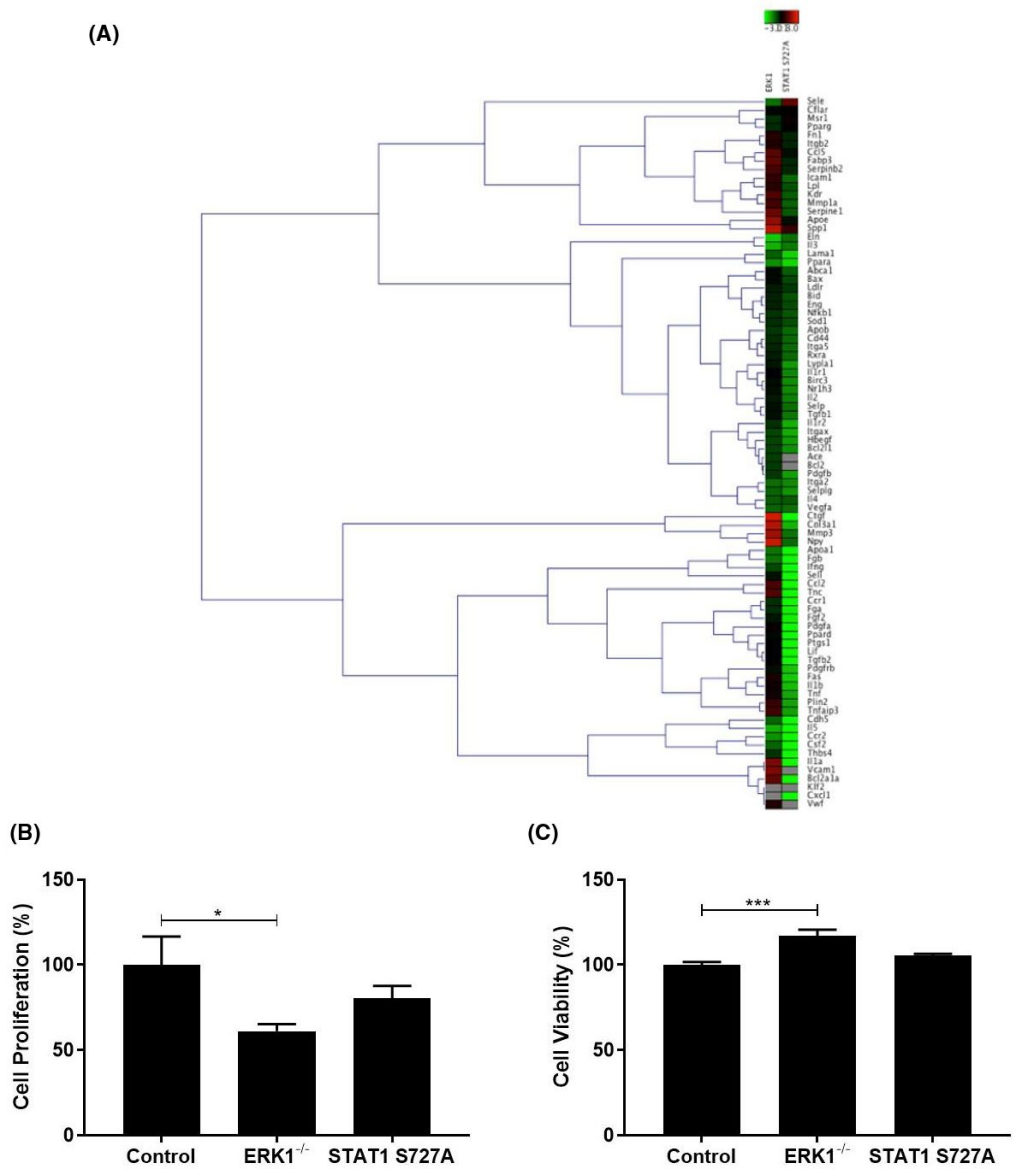
The effect of ERK1 deficiency and STAT1 S727A modification on the expression of atherosclerosis‐associated genes and the proliferation of macrophages. BMDM were isolated from C57BL/6J (Control), ERK1^−/−^, or STAT1 S727A mice and cultured for 24 h. (A) RNA was isolated and gene expression analyzed using RT^2^ Profiler PCR Arrays as described in Materials and Methods. The heat maps present the log2 fold change in gene expression in BMDM from ERK1^−/−^ or STAT1 S727A mice following normalization to the C57BL/6J control, which was arbitrarily assigned as 1. The Genesis software was used to assess gene expression signals and clustering with a scale of color changes on the top of the heat map showing the intensity of gene expression. The full list of genes together with the changes in their expression are shown in Tables S1 and S2. (B, C) Media were removed from the cells and used to determine cell viability by following LDH release (C) with the remaining cells subjected to the crystal violet assay to assess proliferation (B). The changes in BMDM from ERK1^−/−^ or STAT1 S727A mice were normalized to the control, which was arbitrarily assigned as 100%. The data (mean ± SEM) are from four independent experiments. Statistical analysis was performed using a one‐way ANOVA with Tukey's post hoc test (*p ≤ .05; ***p ≤ .001)

The changes in the expression of all the genes in the arrays in BMDM from ERK1^−/−^ and STAT1 S727A mice when compared to the control C57BL/6J mice are shown in Tables S1 and S2, respectively. Figure 1A shows a heat map of hierarchical clustering of gene transcript expression patterns in BMDM from ERK1^−/−^ and STAT1 S727A mice compared to the control. Table 1 displays a summary of the gene expression changes that were significant (*p* ≤ .05) or showed a trend (*p* values between .05 and .1) according to their function. The attenuated expression of *Abca1* and *Mcp‐1* (also called *Ccr2*) genes in BMDM macrophages from STAT1 S727A mice was confirmed by independent conventional RT‐qPCR (Figure S1B), thereby providing additional validation to the findings from the PCR arrays.

**TABLE 1:** The effect of ERK1 deficiency and STAT1 S727A modification on the expression of key atherosclerosis‐associated genes according to function

Gene function	ERK1^−/−^	STAT1 S727A	Common genes
Stress responses (e.g., inflammatory responses, response to pests, pathogens, or parasites)	*Ifng*↓; *Il4*↓; (*Ccr2*↓); (Spp1↑)	*Ccl2*↓; *Ccr1*↓; *Ccr2*↓; *Cxcl1*↓; *Ifng*↓; *Il1a*↓; *Il1b*↓; *Il1r2*↓; *Tgfb1*↓; *Tnf*↓; (*Il1r1*↓); (*Il2*↓);	*Ccr2*; *Ifng*
Apoptosis	(*Bcl2*↓)	*Bcl2*↓; *Bcl2a1a*↓; *Bcl2l1*↓; *Fas*↓; *Tnfaip3*↓; (*Bax*↓)	*Bcl2*
Blood coagulation and circulation		*Ptgs1*↓, *Vwf*↓	
Cell adhesion molecules	*Cdh5*↓; *Itga2*↓; *Lama1*↓; *Sele*↓; *Selplg*↓; (*Sell*↓)	*Cdh5*↓; *Itga5*↓; *Itgax*↓; *Lama1*↓; *Sell*↓; *Selplg*↓; *Thbs4*↓; *Vcam1*↓; (*Itga2*↓); (*Cd44*↓)	*Cdh5*; *Itga2*; *Lama1*; *Sell*; *Selplg*
Extracellular matrix molecules	*Ace*↓; *Eln*↓; *Fgb*↓; *Npy*↑; (Col3a1↑)	*Ace*↓; *Fga*↓; *Fgb*↓; *Mmp1a*↓; *Tnc*↓; (*Mmp*3↓)	*Ace*; *Fgb*
Lipid transport and metabolism	*Apoa1*↓; (Apob↓)	*Abca1*↓; *Apoa1*↓; *Lypla1*↓; *Plin2*↓; (Lpl↓)	*Apoa1*
Cell growth and proliferation	*Csf2*↓; *Hbegf*↓; *Il3*↓; *Il5*↓; *Vegfa*↓	*Csf2*↓; *Ctgf*↓; *Fgf2*↓; *Hbegf*↓; *Il3*↓; *Il5*↓; *Lif*↓; *Pdgfa*↓; *Pdgfb*↓; *Pdgfrb*↓; *Tgfb2*↓; *Vegfa*↓	*Csf2*; *Hbegf*; *Il3*; *Il5*; *Vegfa*
Transcriptional regulation	*Ppara*↓	*Nr1h3*↓; *Ppara*↓; *Ppard*↓; (*Nfkb1*↓); (*Rxra*↓)	*Ppara*

Note

↑ indicates induction or ↓ reduction in expression with those in parenthesis showing trends (*p* values between .05 and .1).

Abbreviations: *Abca1*, ATP‐binding cassette, subfamily A (ABC1), member 1; *Ace*, angiotensin I converting enzyme (peptidyl‐dipeptidase A) 1; *Apoa1*, apolipoprotein A‐I; *ApoB*, apolipoprotein B; *Bcl2*, B cell leukemia/lymphoma 1; *Bcl2a1a* (Bfl‐1, A1), B cell leukemia/lymphoma 2 related protein A1a; *Bcl2I1* (Bcl‐XL), BCL2‐like 1; *Ccl2* (MCP‐1), chemokine (C‐C motif) ligand 2; *Ccr1*, chemokine (C‐C motif) receptor 1; *Ccr2*, chemokine (C‐C motif) receptor 2; *Cdh5*, cadherin 5; *Csf2* (GMCSF), colony stimulating factor 2 (granulocyte‐macrophage); *Ctgf*, connective tissue growth factor; *Cxcl1* (Gro1), chemokine (C‐X‐C motif) ligand 1; *Eln*, elastin; Eng1 (Evi‐1), endoglin; *Fas* (TNFRSF6), TNF receptor superfamily member 6; *Fga*, fibrinogen α chain; *Fgb*, fibrinogen β chain; *Fgf2* (bFGF), fibroblast growth factor 2; Hbegf (Dtr), heparin‐binding EGF‐like growth factor; *Ifng*, interferon‐γ; *Il1a*, interleukin‐1α; *Il1b*, interleukin‐1β; *Il1r1*, interleukin 1 receptor, type I; *Il1r2*, interleukin 1 receptor, type II; *Il2*, interleukin‐2; *Il3*, interleukin‐3; *Il4*, interleukin‐4; *Il5*, interleukin‐5; *Itga2*, integrin α2; *Itga5*, integrin α5 (fibronectin receptor α); *Itgax*, integrin α X; *Lama1*, laminin α1; *Lif*, leukemia inhibitory factor; *Lpl*, lipoprotein lipase; *Lypla1*, lysophospholipase 1; *Mmp1a*, matrix metallopeptidase 1a (interstitial collagenase); *Mmp3*, matrix metallopeptidase 3; *Nfkb1*, nuclear factor of kappa light polypeptide gene enhancer in B cells 1, p105; *Npy*, neuropeptide Y; *Nr1h3*, nuclear receptor subfamily 1, h group H, member 3; *Pdgfa*, platelet derived growth factor α; *Pdgfb*, platelet derived growth factor, B polypeptide; *Pdgfrb*, platelet derived growth factor receptor, beta polypeptide; *Plin2*, perlipin 2; *Ppara*, peroxisome proliferator activated receptor α; *Ppard*, peroxisome proliferator activated receptor δ; *Ptgs1* (COX1), prostaglandin‐endoperoxide synthase 1; *Rxra*, retinoid X receptor α; *Sele*, selectin, endothelial cells; *Sell* (LECAM‐1), selectin, lymphocyte; *Selpg* (P‐Selectin), selectin, platelet (p‐selectin) ligand; *Spp1*, secreted phosphoprotein 1; *Tgfb1*, transforming growth factor‐β1; *Tgfb2*, transforming growth factor‐β2; *Thbs4*, thrombospondin 4; *Tnc*, tenascin C; *Tnf*, tumor necrosis factor; *Tnfaip3*, tumor necrosis factor, alpha‐induced protein 3; *Vcam1*, vascular cell adhesion molecule 1; *Vegfa*, vascular endothelial growth factor A; *Vwf*, Von Willebrand factor.

As shown in Table 1 and Table S1, the ERK1 deficiency resulted in a borderline significant increase in the expression of one gene (*Npy*, *p* = .050) and a significant decrease in the expression of 17 genes: *Ace* (*p* = .044); *Apoa1* (*p* = .003); *Cdh5* (*p* = .002); *Csf2* (*p* < .001); *Eln* (*p* < .001); *Fgb* (*p* = .001); *Hbegf* (*p* = .043); *Ifng* (*p* = .039); *Il3* (*p* < .001); *Il4* (*p* = .007); *Il5* (*p* < .001); *Itga2* (*p* < .001); *Lama1* (*p* = .008); *Ppara* (*p* < .001); *Sele* (*p* = .009); *Selplg* (*p* = .019); and *Vegfa* (*p* = .004). In addition, there was a trend toward increased expression of *Col3a1* (*p* = .095) and *Spp1* (*p* = .062) and a trend toward reduced expression of *Apob* (*p* = .094), *Bcl2* (*p* = .086), *Ccr2* (*p* = .087), and *Sell* (*p* = .071). The expression of 49 genes was significantly attenuated by STAT1 S727A modification: *Abca1* (*p* = .021); *Ace* (*p* < .001); *Apoa1* (*p* < .001); *Bcl2* (*p* < .001); *Bcl2a1a* (*p* < .001); *Bcl2l1* (*p* = .006); Ccl2 (*p* = .002); *Ccr1* (*p* = .007); *Ccr2* (*p* < .001); *Cdh5* (*p* = .014); *Csf2* (*p* < .001); *Ctgf* (*p* < .001); *Cxcl1* (*p* < .001); *Fas* (*p* = .003); *Fga* (*p* < .001); *Fgb* (*p* = .009); *Fgf2* (*p* = .009); *Hbegf* (*p* = .014); *Ifng* (*p* = .002); *Il1a* (*p* < .001); *Il1b* (*p* = .003); *Il1r2* (*p* = .012); *Il3* (*p* = .042); *Il5* (*p* < .001); *Itga5* (*p* = .016); *Itgax* (*p* = .006); *Lama1* (*p* = .020); *Lif* (*p* = .002); *Lypla1* (*p* = .003); *Mmp1a* (*p* = .035); *Nr1h3* (*p* = .019); *Pdgfa* (*p* = .001); *Pdgfb* (*p* = .001); *Pdgfrb* (*p* = .006); *Plin2* (*p* = .004); *Ppara* (*p* = .004); *Ppard* (*p* < .001); *Ptgs1* (*p* < .001); *Sell* (*p* < .001); *Selpg* (*p* = .003); *Tgfb1* (*p* = .017); *Tgfb2* (*p* = .001); *Thbs4* (*p* < .001); *Tnc* (*p* < .001); *Tnf* (*p* = .009); *Tnfaip3* (*p* = .044); *Vcam1* (*p* < .001); *Vegfa* (*p* = .049); and *Vwf* (*p* < .001). In addition, there was a trend toward reduced expression of nine genes: *Bax* (*p* = .093); *Cd44* (*p* = .069); *Il1r1* (*p* = .061); *Il2* (*p* = .057); *Itga2* (*p* = .059); *Lpl* (*p* = .095); *Mmp3* (*p* = .056); *Nfkb1* (*p* = .064); and *Rxra* (*p* = .053).

A large number of gene expression changes produced by the ERK1 deficiency and/or the STAT1 S727A modification were anti‐atherogenic and included decreased expression of key genes implicated in inflammation and stress responses (e.g., *Ccl2*, *Ccr1*, *Ccr2*, *Cxcl1*, *Ifng*, *Il1a*, *Il1b*, *Il1r1*, *Il1r2*, and *Tnf*), cell adhesion and migration (e.g., *Cdh5*, *Itga2*, *Itga5*, *Itgax*, *Lama1*, *Sell*, *Sele*, *Selpg, Thbs4, and Vcam1*), cell growth and proliferation (e.g., *Csf2*, *Vegfa*, *Pdgfa, Pdgfb, and Pdgfrb*), apoptosis (e.g., *Fas* and *Tnfaip3*), lipid transport and metabolism (e.g., *Lpl*), proteolysis (e.g., *Ace*, *Mmp1a* and *Mmp3*), and transcriptional regulation (e.g., *Nfkb1*). However, a few gene expression changes were pro‐atherogenic (e.g., *Apoa1*, *Abca1*, *Il5*, *Tgfb1*, *Nr1h3*, *Ppara*, and *Ppard*). Overall, there were 17 common genes whose expression was affected by both the ERK1 deficiency or the STAT1 S727A modification (Table 1; *Ace*, *Apoa1*, *Ccr2*, *Csf2*, *Bcl2*, *Cdh5*, *Fgb*, *Hbegf*, *Il3*, *Il5*, *Ifng*, *Itga2*, *Lama1*, *Ppara*, *Sell*, *Selpg*, and *Vegfa*).

### 3.2 The effect of deficiency of ERK1 and STAT1 S727 phosphorylation on key macrophage processes associated with atherosclerosis

Because the expression of a large number of pro‐atherogenic genes was attenuated by the ERK1^−/−^ and STAT1 S727A genetic modifications, their effects on several macrophage processes associated with atherosclerosis was investigated using BMDM from C57BL/6J, ERK1^−/−^, and STAT1 S727A mice. Macrophage proliferation, which is increasingly being implicated in the pathogenesis of atherosclerosis,^27^ was first determined. The proliferation of BMDM, as determined using the crystal violet assay, was significantly decreased for ERK1^−/−^ mice when compared to C57BL/6J mice (*p* = .040) whereas no significant changes were seen for STAT1 S727A mice (Figure 1B). The reduction in cell proliferation with BMDM from ERK1^−/−^ mice was not because of a decrease in cell viability as determined by following the release of the LDH enzyme (Figure 1C).

**FIGURE 2:**
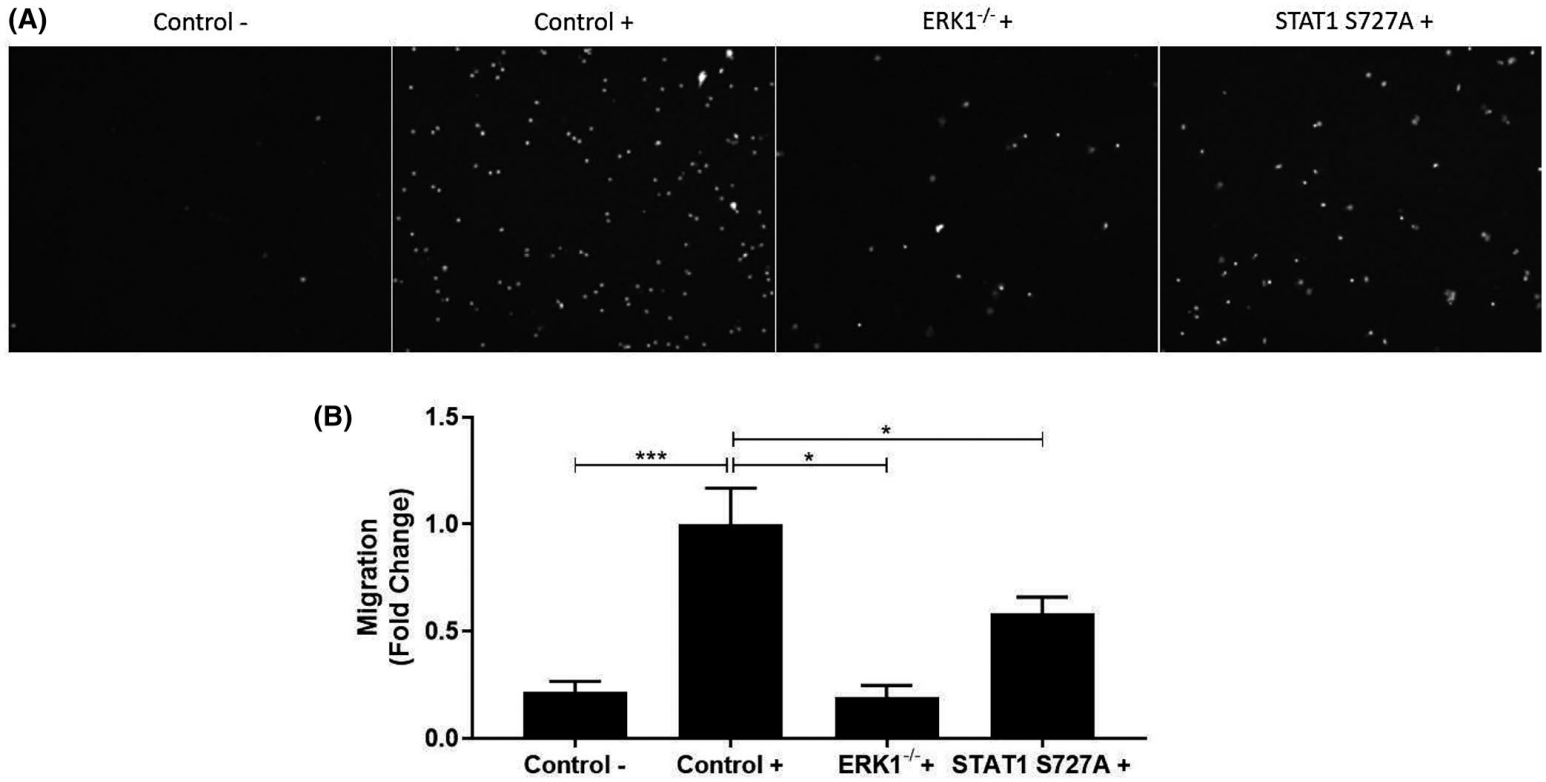
The ERK1 deficiency and STAT1 S727A modification attenuate chemokine‐driven macrophage migration. Migration of BMDM from C57BL/6J (Control), ERK1^−/−^, or STAT1 S727A mice in response to the chemokine MCP‐1 (20 ng/ml, +) was carried out as described in Materials and Methods. Migration of BMDM from control mice in the absence of MCP‐1 (−) was also analyzed for comparative purposes. The migration of the cells was assessed by counting the migrated cells using a fluorescence microscope after staining them with DAPI. Representative images are presented in panel A with graphs in panel B showing fold change in migration (mean ± SEM) from four independent experiments (the value in Control + has been arbitrarily assigned as 1). Statistical analysis was performed using a one‐way ANOVA with Tukey's post hoc test (*p ≤ .05; ***p ≤ .001)

Many of the gene expression changes produced by the genetic modifications relate to proteins that play key roles in cell migration and adhesion (Table 1). In order to delineate the functional consequences of such regulation of gene expression, the migration of macrophages in response to the key chemokine MCP‐1^1^ was therefore determined. As shown in Figure 2, the migration of BMDM from control C57BL/6J mice was significantly induced by MCP‐1 (*p* < .001) and this was attenuated by the ERK1 deficiency (*p* = .022) or STAT1 S727A modification (*p* = .039).

**FIGURE 3:**
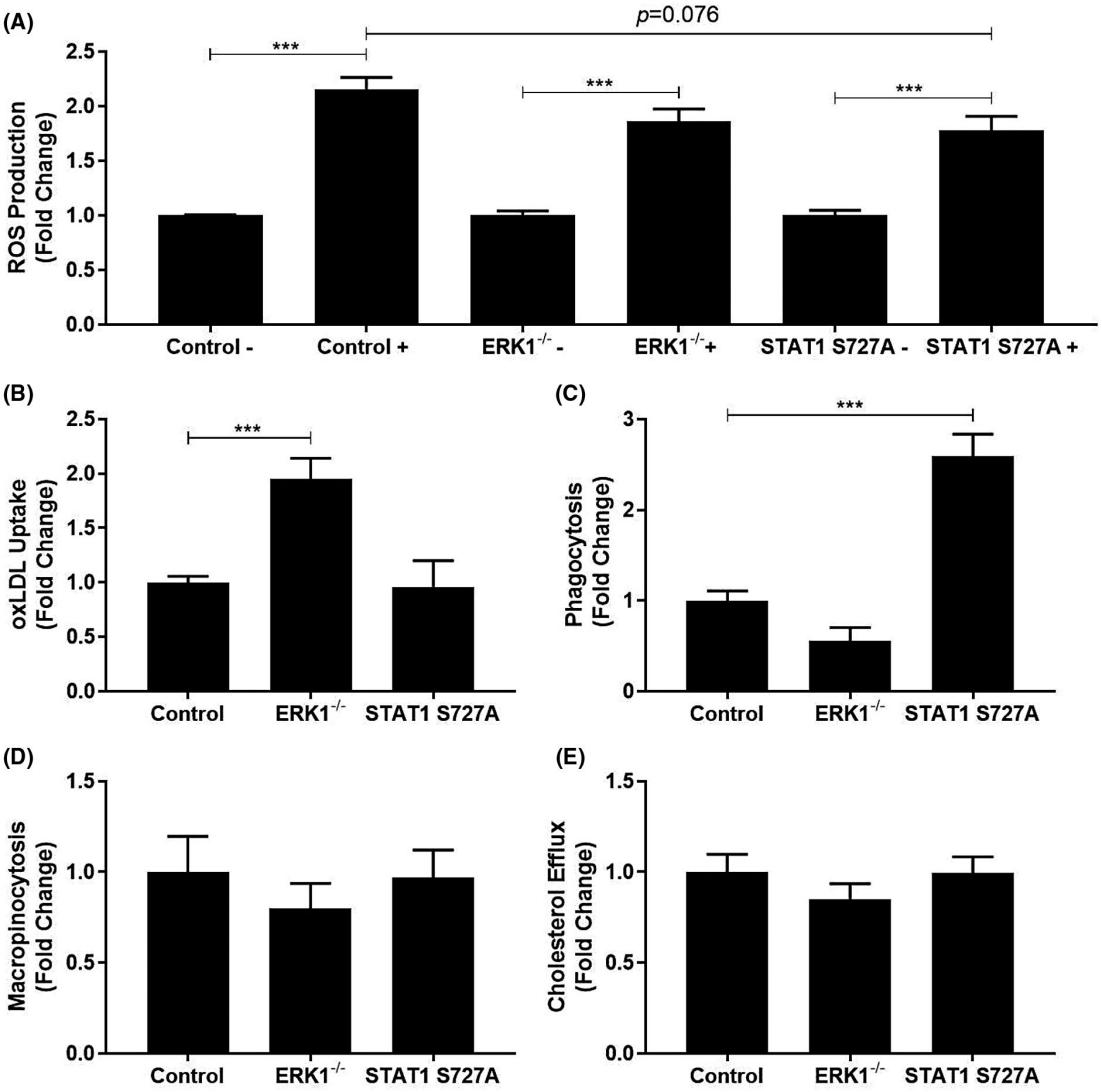
The effect of ERK1 deficiency and STAT1 S727A modification on key macrophage processes associated with atherosclerosis. BMDM were isolated from C57BL/6J (Control), ERK1^−/−^, or STAT1 S727A mice. (A) ROS production was stimulated by incubation with 50 μM TBHP for 3 h (+). BMDM from corresponding mice without any treatment with TBHP were also included for comparative purposes (−). Fluorescence was measured at 495 nm and 520 nm for excitation and emission spectra, respectively. Graph shows mean ± SEM from three independent experiments where ROS production in cells in the absence of any TBHP stimulation has been arbitrarily assigned as 1. (B) The cells were treated for 24 h with Dil‐oxLDL (5 μg/ml) and the uptake assessed by flow cytometry. Graph shows mean ± SEM from three independent experiments with Dil‐oxLDL uptake in BMDM from control mice arbitrarily assigned as 1. (C) Phagocytosis was monitored by the uptake of fluorescently labeled *Escherichia coli* strain K‐12 following 2 h treatment using a Vybrant^TM^ Phagocytosis Assay Kit. Graph shows mean ± SEM from four independent experiments with phagocytosis in BMDM from control mice arbitrarily assigned as 1. (D) Macropinocytosis was assessed by determining the uptake of LY (100 μg/ml) after 24 h incubation followed by flow cytometry. Graph shows mean ± SEM from three independent experiments with macropinocytosis in BMDM from control mice arbitrarily assigned as 1. (E) Macrophages were first converted into foam cells by incubation with 0.5 μCi/ml [^14^C]‐cholesterol and 25 μg/ml AcLDL for 24 h. Cholesterol efflux following incubation for 24 h with 10 μg/ml ApoA1 acceptor was then determined by scintillation counting as described in Materials and Methods. Graph shows mean ± SEM from five independent experiments with efflux in BMDM from control mice arbitrarily assigned as 1. In all cases, statistical analysis was performed using a one‐way ANOVA with Tukey's post hoc test (***p ≤ .001)

In terms of parameters related to macrophage cholesterol homeostasis, a key process in atherogenesis,^2^ ROS production is important for the oxidation of LDL along with endothelial cell dysfunction.^28^ The effect of the genetic modifications on tert‐butyl hydroperoxide (TBHP)‐induced levels of ROS was therefore determined. As shown in Figure 3A, the significant TBHP‐induced ROS production in BMDM from control mice (*p* < .001) was not affected by the ERK1 deficiency whereas there was a trend toward reduction in BMDM from STAT1 S727A mice (*p* = .076). For the uptake of oxLDL, there was no effect in BMDM from STAT1 S727A mice when compared to the control (Figure 3B). In contrast, there was a significant increase with BMDM from ERK1^−/−^ mice (Figure 3B; *p* < .001). Phagocytosis and macropinocytosis also contribute to macrophage foam cell formation.^2^ ERK1 deficiency had no effect on phagocytosis whereas this was significantly increased with the STAT1 S727A modification (Figure 3C; *p* < .001). In contrast, no significant changes were observed for macropinocytosis, as judged by the uptake of the dye LY^22^ (Figure 3D), or the efflux of cholesterol from foam cells (Figure 3E).

### 3.3 The effect of deficiency of ERK1 and STAT1 S727 phosphorylation on plasma levels of total cholesterol, LDL/VLDL, HDL, and TG

For studies on atherosclerosis development and disease‐associated parameters, LDLR^−/−^, LDLR^−/−^/ERK1^−/−^, and LDLR^−/−^/STAT1 S727A mice were fed a high fat diet (HFD) for 12 and 24 weeks. Table S3 shows the outcome of analysis of organ weights and plasma lipid profile. The only significant changes in organ weight were observed following 24 weeks of HFD feeding; decrease in heart weight in LDLR^−/−^/STAT1 S727A animals (*p* < .001) and reduction in the weight of spleen in LDLR^−/−^/ERK1^−/−^ mice (*p* = .032) and LDLR^−/−^/STAT1 S727A mice (*p* < .001) compared to the LDLR^−/−^ controls. In the case of lipid profile at 12 weeks of HFD feeding, the only significant changes were an increase in plasma levels of TG in LDLR^−/−^/ERK1^−/−^ mice (*p* = .001) and LDLR^−/−^/STAT1 S727A mice (*p* = .039) when compared to the LDLR^−/−^ controls. At 24 weeks of HFD feeding, the only significant changes were an increase in plasma LDL/VLDL and TG in LDLR^−/−^/ERK1^−/−^ mice when compared to the LDLR^−/−^ controls (*p* = .011 and *p* = .007, respectively). Overall, therefore, the only common significant change seen in LDLR^−/−^/ERK1^−/−^ mice and LDLR^−/−^/STAT1 S727A mice was an increase in TG levels at 12 weeks.

### 3.4 The effect of ERK1 and STAT1 S727 phosphorylation deficiency on cardiac hypertrophy index

**FIGURE 4:**
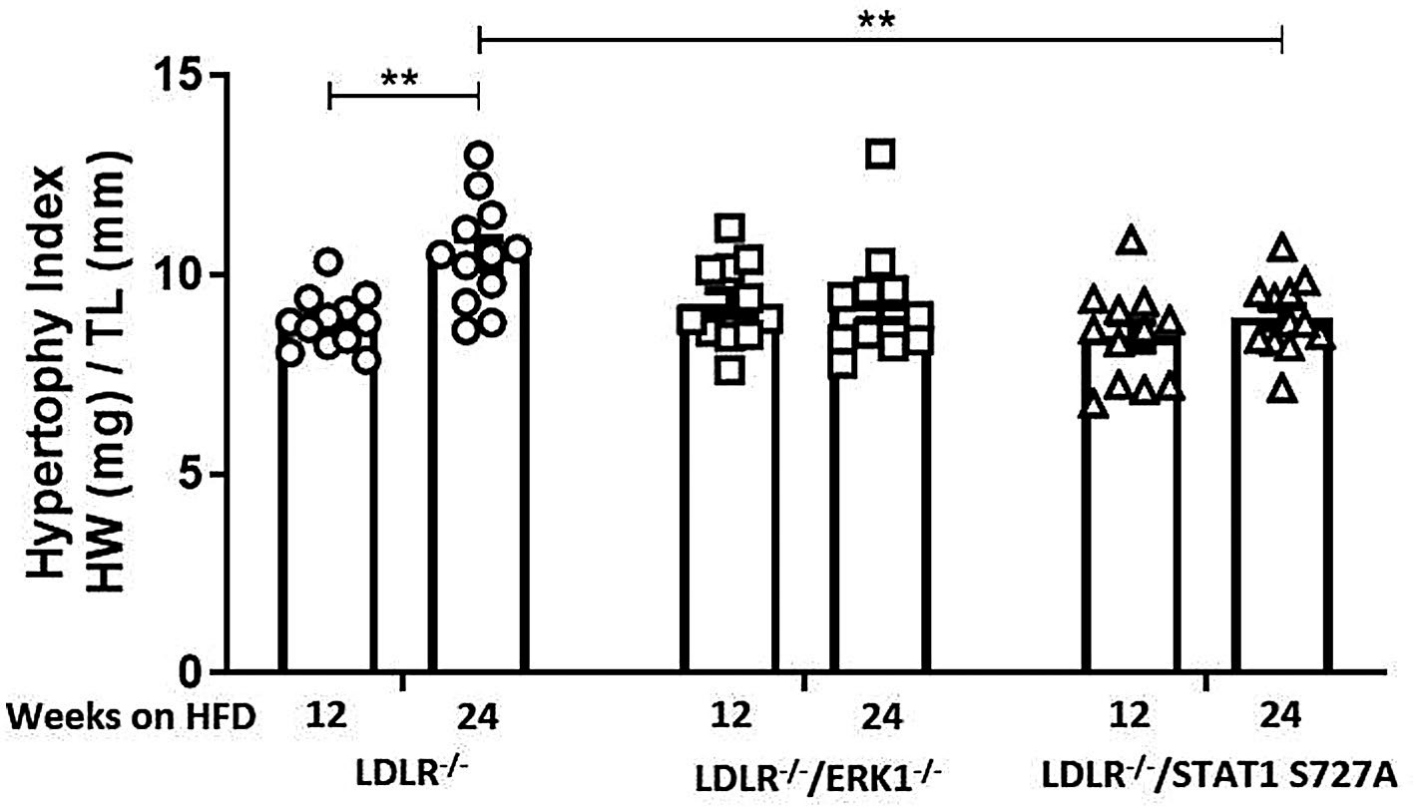
The effect of ERK1 deficiency and STAT1 S727A modification on cardiac hypertrophy index. LDLR^−/−^, LDLR^−/−^/ERK1^−/−^, or LDLR^−/−^/STAT1 S727A mice were fed a HFD for 12‐ or 24‐weeks as indicated. Heart hypertrophy was determined by dividing the heart weight (HW; mg) by the tibia length (TL; mm) for each mice. Graph shows mean ± SEM (*n* = 12, *n* = 12 and *n* = 11, respectively, for LDLR^−/−^, LDLR^−/−^/ERK1^−/−^, or LDLR^−/−^/STAT1 S727A mice fed HFD for 12 weeks and *n* = 12, *n* = 12 and *n* = 11, respectively, for LDLR^‐/^, LDLR^−/−^/ERK1^−/−^ or LDLR^−/−^/STAT1 S727A mice fed HFD for 24 weeks). Statistical analysis was performed using a two‐way ANOVA with Sidak post hoc test (**p ≤ .01)

The cardiac hypertrophic indices were calculated by normalizing whole heart weight (mg) to tibial length (mm) as in previous studies.^29^, ^30^ There was a significant increase in hypertrophic indices of LDLR^−/−^ mice, but not LDLR^−/−^/ERK1^−/−^ and LDLR^−/−^/STAT1 S727 mice, following feeding of HFD for 24 weeks when compared to 12 weeks (Figure 4; *p* = .005). In addition, the hypertrophy index in LDLR^−/−^/STAT1 S727 mice at 24 weeks was significantly decreased when compared to the control LDLR^−/−^ mice (Figure 4; *p* = .008).

### 3.5 The ERK1 deficiency and STAT1 S727A modification decreases plaque lipid content

**FIGURE 5:**
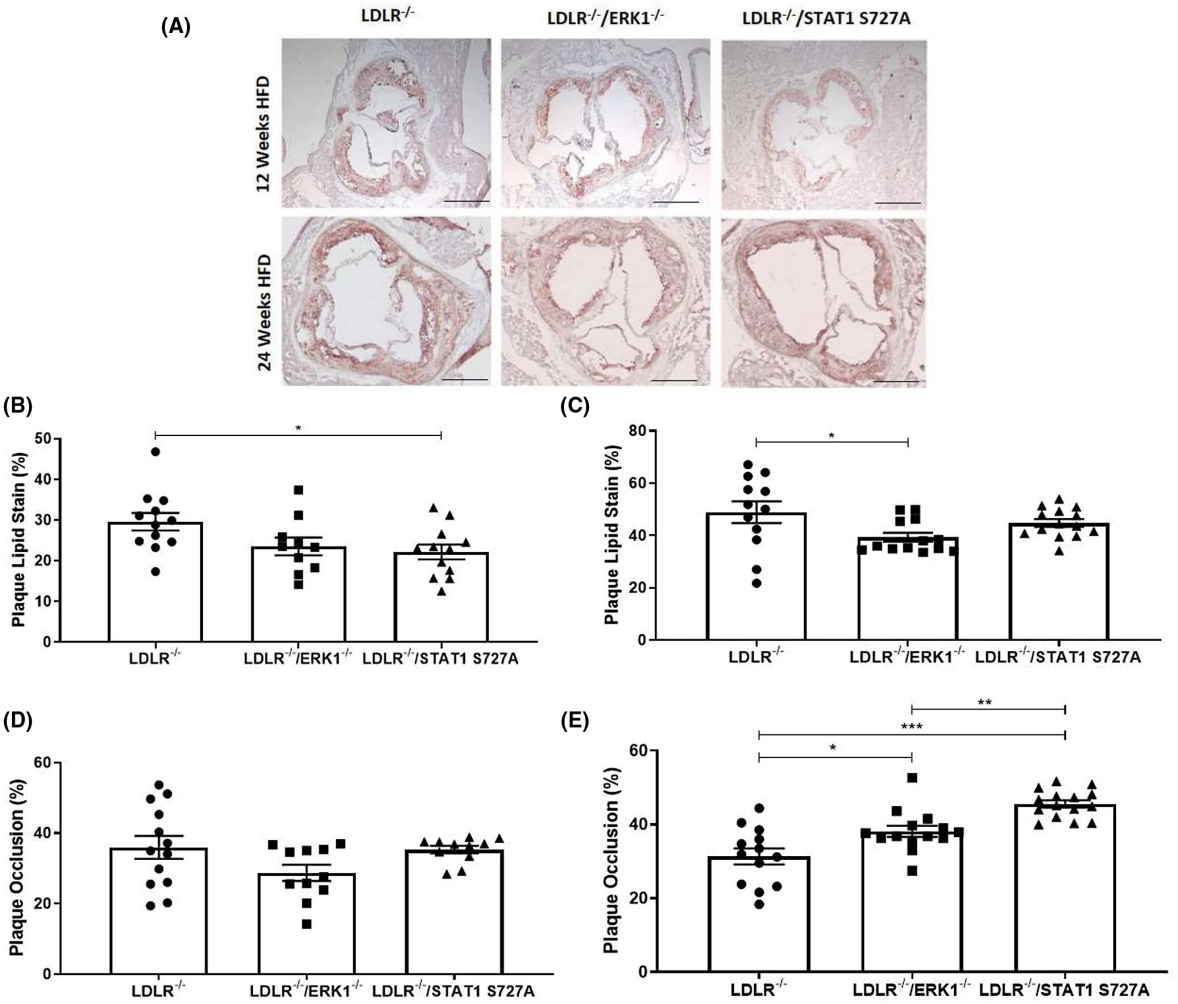
ERK1 deficiency and STAT1 S727A modification impact the lipid content and occlusion of plaques. LDLR^−/−^, LDLR^−/−^/ERK1^−/−^, or LDLR^−/−^/STAT1 S727A mice were fed HFD for 12 or 24 weeks. Sections of the aortic root were stained with Oil red O and hematoxylin counterstain. Representative images are shown in panel A (5× magnification and scale bar of 400 μm). The graphs in panel B‐C show mean ± SEM of lipid content within the plaque [*n* = 12, *n* = 10 and *n* = 12, respectively, for LDLR^−/−^, LDLR^−/−^/ERK1^−/−^, or LDLR^−/−^/STAT1 S727A mice fed HFD for 12 weeks (B) and *n* = 12, *n* = 13, and *n* = 14, respectively, for LDLR^−/−^, LDLR^−/−^/ERK1^−/−^, or LDLR^−/−^/STAT1 S727A mice fed HFD for 24 weeks (C)]. The graphs in panel D‐E shows mean ± SEM of percentage plaque occlusion [*n* = 13, *n* = 11, and *n* = 11, respectively, for LDLR^−/−^, LDLR^−/−^/ERK1^−/−^, or LDLR^−/−^/STAT1 S727A mice fed HFD for 12 weeks (D) and *n* = 13, *n* = 14, and *n* = 15, respectively, for LDLR^−/−^, LDLR^−/−^/ERK1^−/−^, or LDLR^−/−^/STAT1 S727A mice fed HFD for 24 weeks (E)]. Statistical analysis was performed using a one‐way ANOVA with Tukey's post hoc test (*p ≤ .05; **p ≤ .01; ***p ≤ .001)

Oil red O and hematoxylin staining was carried out to determine the percentage lipid content of the aortic root plaques (Figure 5A) given that high lipid content is associated with severe atherosclerosis.^28^ There was a significant reduction in plaque lipid levels of the aortic root following feeding of HFD for 12 weeks for LDLR^−/−^/STAT1 S727A mice compared to the control LDLR^−/−^ mice (*p* = .033; Figure 5B). There was no significant change after 12 weeks of HFD feeding for the LDLR^−/−^/ERK1^−/−^ group and fell outside the trend range of *p* values (*p* = .117; Figure 5B). However, following 24 weeks of HFD feeding, the plaque lipid content in the LDLR^−/−^/ERK1^−/−^ mice was significantly decreased (*p* = .038) when compared to the LDLR^−/−^ control mice (Figure 5C). No such significant changes were seen in the LDLR^−/−^/STAT1 S727A mice following feeding of HFD for 24 weeks when compared to the LDLR^−/−^ control (Figure 5C).

The percentage occlusion of the total vessel lumen was also determined (i.e., degree of luminal obstruction caused by plaque). As shown in Figure 5D, no significant changes were observed following 12 weeks of HFD feeding for both LDLR^−/−^/ERK1^−/−^ and LDLR^−/−^/STAT1 S727A mice when compared to the LDLR^−/−^ control mice. However, a significant increase in plaque occlusion was observed following 24 weeks feeding HFD in LDLR^−/−^/ERK1^−/−^ mice (*p* = .012) and LDLR^−/−^/STAT1 S727A mice (*p* < .001) when compared to the LDLR^−/−^ control mice (Figure 5E). In addition, a significant increase was also seen between LDLR^−/−^/STAT1 S727A and LDLR^−/−^/ERK1^−/−^ mice (*p* = .004) (Figure 5E). Overall, these data suggest a potential protective action of deficiency of ERK1 and STAT1 S727 phosphorylation on plaque lipid content with no adverse effects on this parameter following feeding of a HFD for longer duration.

### 3.6 The plaque macrophage content was reduced in LDLR^−/−^/STAT1 S727A mice following 12 weeks feeding of HFD

**FIGURE 6:**
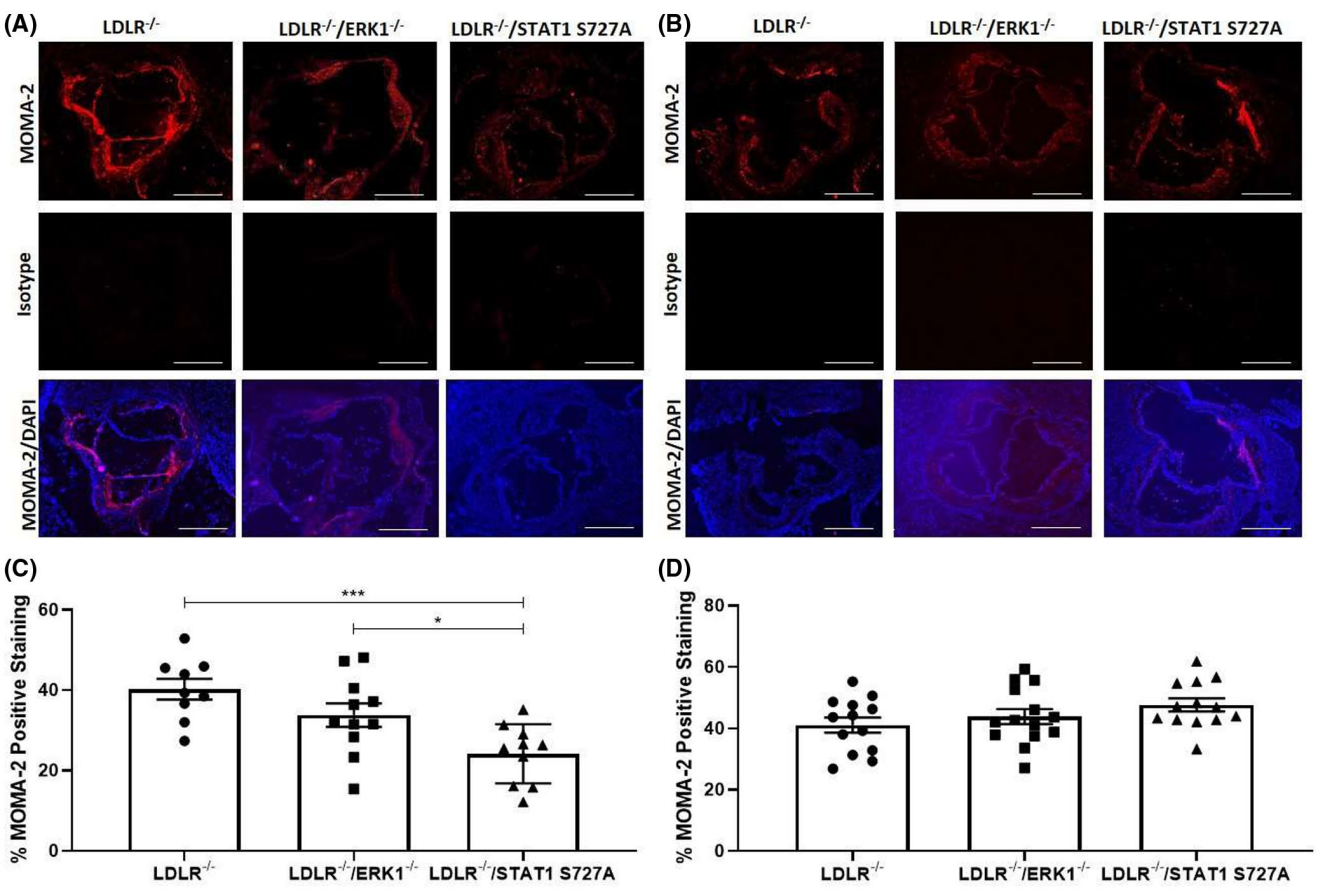
The STAT1 S727A modification attenuates plaque macrophage content following feeding of HFD for 12 weeks. LDLR^−/−^, LDLR^−/−^/ERK1^−/−^, or LDLR^−/−^/STAT1 S727A mice were fed a HFD for 12 or 24 weeks (A, C and B, D, respectively). Sections of the aortic root were stained for MOMA‐2 positive macrophages. Representative images are shown in panels A‐B (5× magnification and scale bar of 400 μm). The graphs show mean ± SEM of the MOMA‐2 macrophage content within the plaque [*n* = 9, *n* = 11, and *n* = 10, respectively, for LDLR^−/−^, LDLR^−/−^/ERK1^−/−^, or LDLR^−/−^/STAT1 S727A mice fed HFD for 12 weeks (C) and *n* = 13, *n* = 14, and *n* = 13, respectively for LDLR^−/−^, LDLR^−/−^/ERK1^−/−^, or LDLR^−/−^/STAT1 S727A mice fed HFD for 24 weeks (D)]. Statistical analysis was performed using a one‐way ANOVA with Tukey's post hoc test (*p ≤ .05; ***p ≤ .001)

Macrophage infiltration is a key marker for plaque inflammation^1^, ^2^, ^28^ and was therefore determined in aortic root plaques by immunofluorescent staining using MOMA‐2 antibody (Figure 6). There was a significant reduction in percentage plaque area of MOMA‐2 staining in the aortic root following feeding of a HFD for 12 weeks in LDLR^−/−^/STAT1 S727A mice when compared to the LDLR^−/−^ control mice (*p* < .001; Figure 6C). In addition, there was also a significant reduction in MOMA‐2 staining in LDLR^−/−^/STAT1 S727A mice compared to LDLR^−/−^/ERK1^−/−^ mice (*p* = .036; Figure 6C) However, no such significant changes in MOMA‐2 staining were seen following feeding of the mice with HFD for 24 weeks (Figure 6D).

### 3.7 The impact of the genetic modifications on plaque CD3+ T‐cell content following feeding of HFD

**FIGURE 7:**
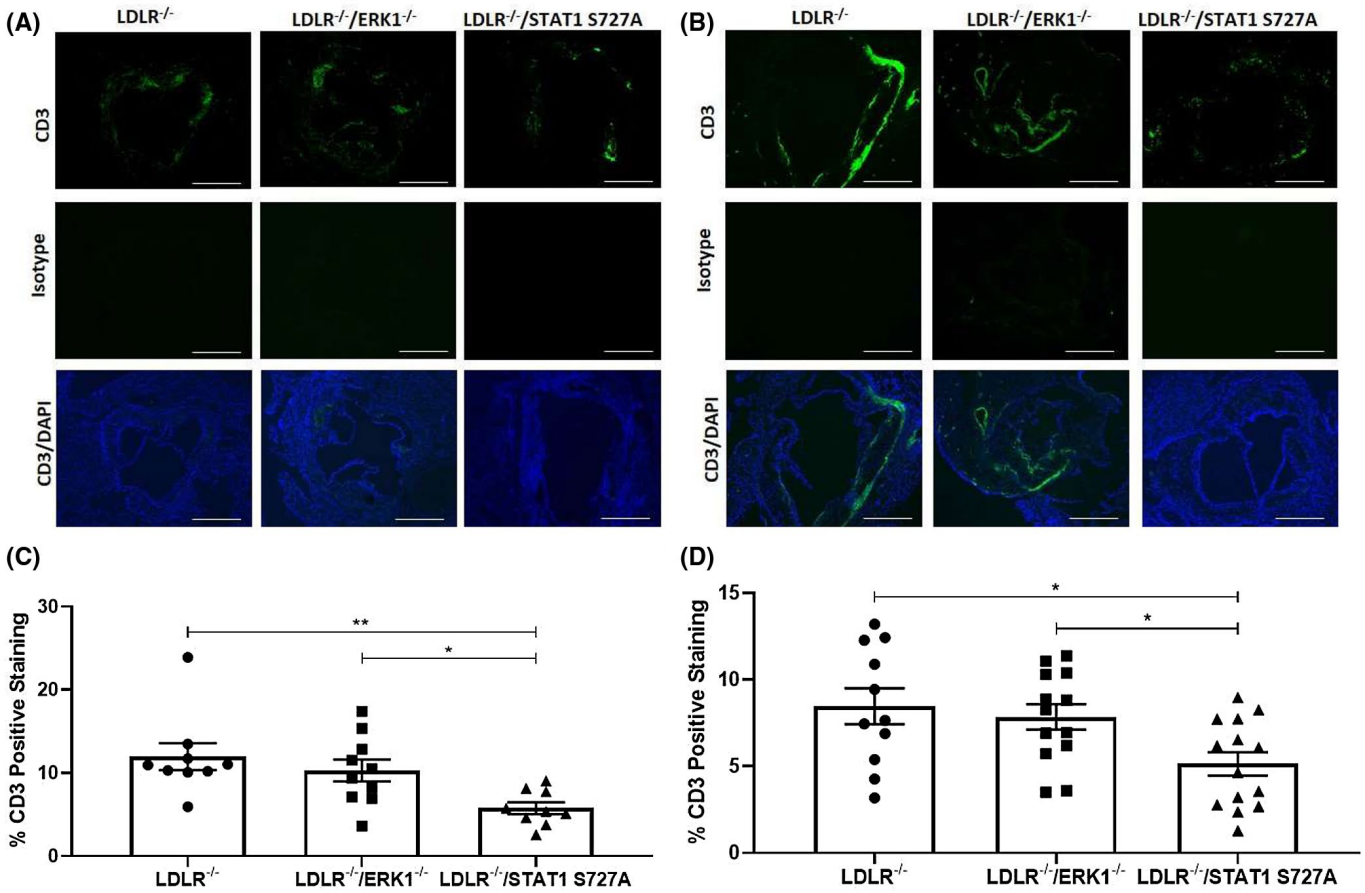
The effect of ERK1 deficiency and STAT1 S727A modification on CD3+ T‐cell content following feeding of HFD. LDLR^−/−^, LDLR^−/−^/ERK1^−/−^, or LDLR^−/−^/STAT1 S727A mice were fed HFD for 12 or 24 weeks (A, C and B, D, respectively). Sections of the aortic root were stained for CD3+ positive T cells. Representative images are shown in panels A‐B (5× magnification and scale bar of 400 μm). The graphs show mean ± SEM of the CD3+ T cells within the plaque [*n* = 9, *n* = 10, and *n* = 9, respectively, for LDLR^−/−^, LDLR^−/−^/ERK1^−/−^, or LDLR^−/−^/STAT1 S727A mice fed HFD for 12 weeks (C) and *n* = 11, *n* = 13, and *n* = 14, respectively, for LDLR^−/−^, LDLR^−/−^/ERK1^−/−^, or LDLR^−/−^/STAT1 S727A mice fed HFD for 24 weeks (D)]. Statistical analysis was performed using a one‐way ANOVA with Tukey's post hoc test on log‐transformed data (*p ≤ .05; **p ≤ .01)

In addition to macrophages, the content of CD3+ T lymphocytes is often indicative of the inflammatory status^1^, ^24^ and was therefore determined (Figure 7). There was a significant reduction of CD3+ T cells in LDLR^−/−^/STAT1 S727A mice but not in LDLR^−/−^/ERK1^−/−^ mice following feeding of a HFD for 12 weeks and 24 weeks when compared to the LDLR^−/−^ controls (*p* = .007 and *p* = .018, respectively; Figure 7C,D). In addition, the content of CD3+ T cells in LDLR^−/−^/STAT1 S727A mice was significantly reduced compared to LDLR^−/−^/ERK1^−/−^ mice at 12 weeks and 24 weeks (*p* = .049 in both cases; Figure 7C,D).

### 3.8 The effect of deficiency of ERK1 and STAT1 S727 phosphorylation on plaque stability

**FIGURE 8:**
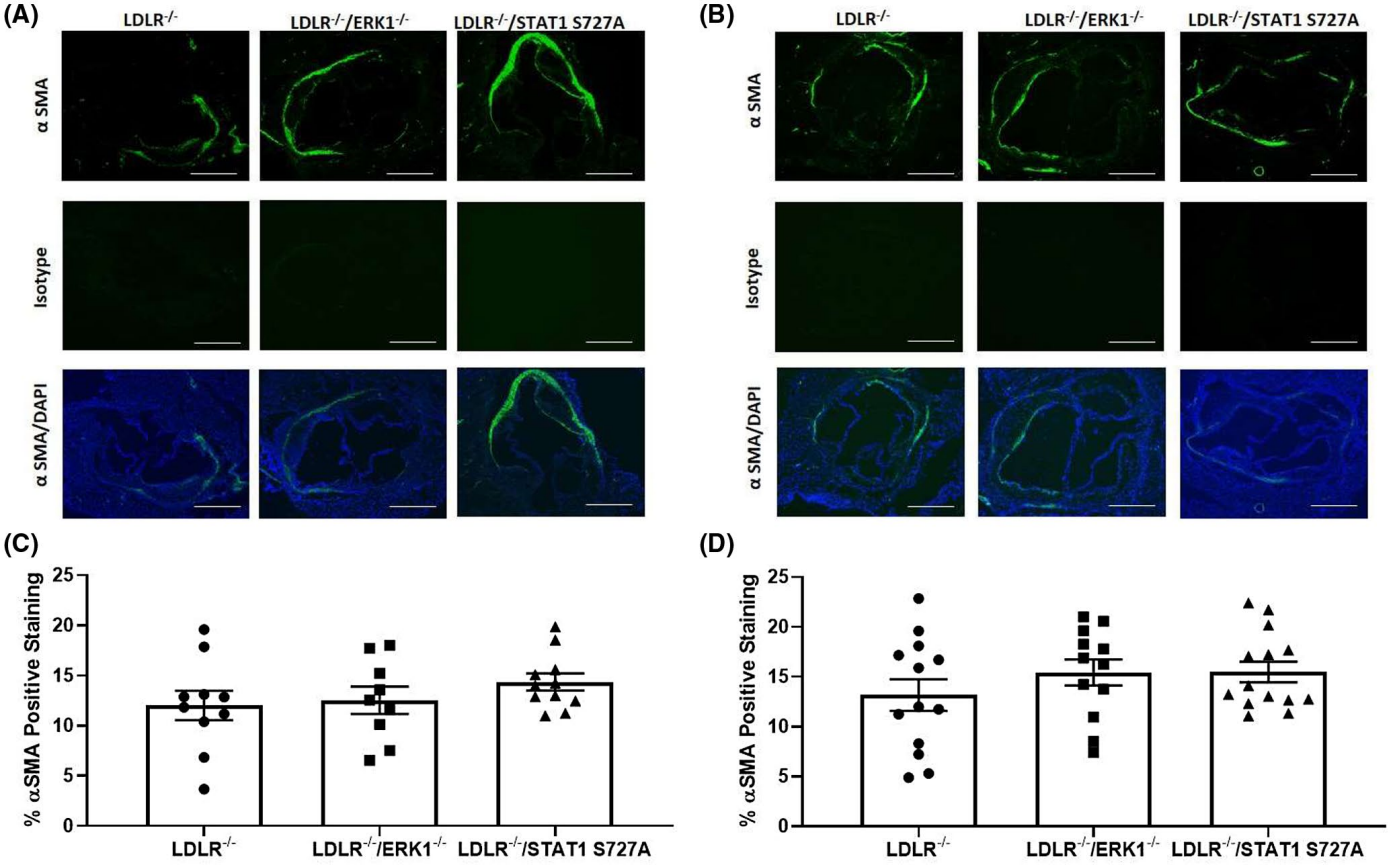
The effect of ERK1 deficiency and STAT1 S727A modification on the smooth muscle cell content of plaques. LDLR^−/−^, LDLR^−/−^/ERK1^−/−^, or LDLR^−/−^/STAT1 S727A mice were fed HFD for 12 or 24 weeks (A, C and B, D, respectively). Sections of the aortic root were stained for smooth muscle cell content. Representative images are shown in panels A‐B (5× magnification and scale bar of 400 μm). The graphs show mean ± SEM of smooth muscle cell content within the plaque [*n* = 10, *n* = 9, and *n* = 11 respectively, for LDLR^−/−^, LDLR^−/−^/ERK1^−/−^, or LDLR^−/−^/STAT1 S727A mice fed HFD for 12 weeks (C) and *n* = 13, *n* = 12, and *n* = 14, respectively, for LDLR^−/−^, LDLR^−/−^/ERK1^−/−^, or LDLR^−/−^/STAT1 S727A mice fed HFD for 24 weeks (D)]. Statistical analysis was performed using a one‐way ANOVA with Tukey's post hoc test

α‐smooth muscle cell actin was used as a marker of vascular smooth muscle cell (VSMC) infiltration into the plaques and thereby plaque stability^24^, ^28^ (Figure 8). There was no significant change in VSMC content in plaques of LDLR^−/−^/ERK1^−/−^ and LDLR^−/−^/STAT1 S727A mice following feeding of HFD for 12 weeks or 24 weeks when compared to control mice (Figure 8). The plaque collagen content was also determined as an additional parameter for the effect of genetic modifications on plaque stability. As shown in Figure S2, the plaque collagen content was not affected in LDLR^−/−^/ERK1^−/−^ and LDLR^−/−^/STAT1 S727A mice following feeding of HFD for 12 weeks or 24 weeks when compared to control mice.

### 3.9 The effect of deficiency of ERK1 and STAT1 S727 phosphorylation on plasma cytokine levels

The plasma levels of several cytokines were determined in LDLR^−/−^, LDLR^−/−^/ERK1^−/−^, and LDLR^−/−^/STAT1 S727A mice following feeding of HFD for 12 weeks; a time point where changes in plaque macrophage and T cell content, indicative of inflammation, were observed in LDLR^−/−^/STAT1 S727A mice (Figures 6 and 7). As shown in Table 2, a significant increase in IL‐2 levels was seen in LDLR^−/−^/STAT1 S727A mice compared to the LDLR^−/−^ control mice (*p* = .026). A trend toward increase (*p* = .094) was also observed in the levels of IL‐5 in LDLR^−/−^/STAT1 S727A mice compared to the LDLR^−/−^ control. No significant changes were observed for the other cytokines analyzed.

**TABLE 2:** Plasma cytokine levels in LDLR^−/−^, LDLR^−/−^/ERK1^−/−^ and LDLR^−/−^/STAT1 S727A mice following feeding of HFD for 12 weeks

Cytokine	LDLR^−/−^(pg/ml)	*N*	LDLR^−/−^/ERK1^−/−^(pg/ml)	*N*	Change	LDLR^−/−^/STAT1 S727A (pg/ml)	*N*	Change
IL‐1β	1.25 ± 0.50	11	0.51 ± 0.15	6	NS	0.54 ± 0.14	5	NS
IL‐2	1.16 ± 0.06	14	1.06 ± 0.06	10	NS	1.42 ± 0.09	12	↑ (*p* = .026)
IL‐4	0.35 ± 0.13	7	0.54 ± 0.28	3	NS	0.20 ± 0.01	2	NS
IL‐5	3.35 ± 0.26	14	2.65 ± 0.20	10	NS	7.58 ± 2.52	12	↑ (*p* = .094)
IL‐6	72.55 ± 22.27	14	42.91 ± 14.41	10	NS	28.95 ± 4.49	11	NS
IFN‐γ	0.39 ± 0.10	14	0.42 ± 0.11	11	NS	0.66 ± 0.09	11	NS
KC/GRO	174.40 ± 18.50	15	129.50 ± 12.20	11	NS	155.00 ± 15.00	12	NS
TNF‐α	16.80 ± 2.10	15	14.27 ± 1.37	11	NS	13.00 ± 1.00	12	NS
IL‐10	17.30 ± 1.00	15	16.59 ± 2.27	10	NS	19.98 ± 0.90	12	NS

Note

Significant or trend of increase (↑) are shown with *p* values in parenthesis. NS, not significant; N, numbers of animals (plasma with undetectable assay readings were removed before statistical analysis).

## 4 DISCUSSION

**FIGURE 9:**
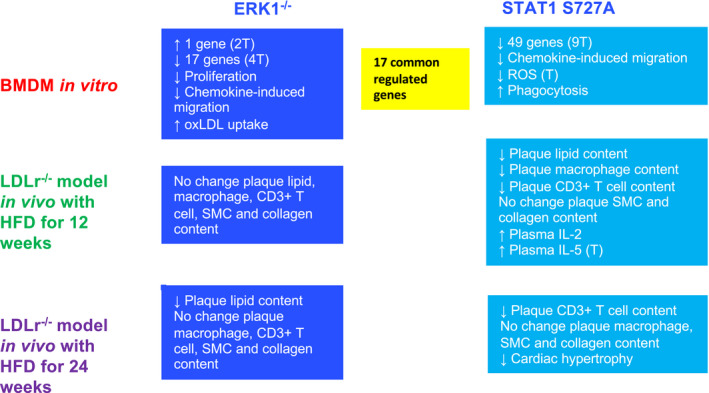
Summary of key outcomes from this study. ↑, Increase; ↓, decrease; T, trend toward significance

Our previous studies using pharmacological inhibitors and RNA interference assays demonstrated a critical role for ERK1/2 in STAT1 S727 phosphorylation associated with IFN‐γ‐mediated regulation of modified LDL uptake and the expression of four key genes implicated in atherosclerosis in human macrophages.^13^ We have carried out here an in‐depth analysis of the role of ERK1 and STAT1 S727 phosphorylation on atherosclerosis using ERK1^−/−^ and STAT1 S727A knock‐in mice and key outcomes are summarized in Figure 9. Atherosclerosis RT² Profiler PCR Array analysis showed that ERK1 deficiency and STAT1 S727A modification produced significant changes in the macrophage expression of 18 and 49 atherosclerosis‐associated genes, respectively (Table 1 and Tables S1–S2). There were 17 common regulated genes, many of which are known to play key roles in the control of cell migration and inflammation (Table 1). In correlation with such gene expression changes, ERK1 deficiency and STAT1 S727A modification was associated with a significant decrease in chemokine‐driven macrophage migration in vitro (Figure 2). In addition, both genetic changes attenuated plaque lipid content in the LDLR^−/−^ model system, albeit at different time points that was not associated with any reduction of plasma lipoproteins or TG levels (Figure 5 and Table S3). However, the STAT1 S727A modification had the most pronounced effect on plaque inflammation and atherosclerosis‐associated parameters. For example, the modification increased macrophage phagocytosis and decreased the expression of a large number of atherosclerosis‐associated genes in vitro, reduced plaque content of macrophages and CD3+ T lymphocytes in vivo, and produced a trend toward increase in plasma levels of the anti‐inflammatory cytokine IL‐5 (Figures 3, 6, and 7, Tables 1 and 2). These results therefore provide novel insights into the atherogenic actions of STAT1 S727 phosphorylation and inform on its potential as a promising therapeutic target.

The in vitro studies were focused on macrophages because of our previous research on ERK1/2 and STAT1 serine 727 phosphorylation on these cells and as they play pivotal roles at all stages of atherosclerosis.^1^, ^2^, ^13^ However, the global nature of deficiency in the models used does not rule out that the observed changes in lesions/atherosclerosis are related at least in part to other cell types. Hence future studies should seek to investigate the impact of ERK1 deficiency and STAT1 serine 727 phosphorylation on key atherosclerosis‐associated processes in endothelial cells, smooth muscle cells, and other immune cells.

Macrophage proliferation and migration play important roles in the pathogenesis of atherosclerosis.^27^, ^31^ Thus, local proliferation has been found to dominate lesional macrophage accumulation in atherosclerosis.^27^ A differential effect was seen on macrophage proliferation with significant inhibition by ERK1 deficiency but not STAT1 S727A modification (Figure 1). The exact reason(s) for such a difference is currently unclear but suggests that the pro‐atherogenic actions of ERK1 are in part mediated via promotion of macrophage proliferation. Plaque macrophages generally have reduced ability to migrate that impedes resolution of inflammation and promotes progression of atherosclerotic lesions to more complex, rupture‐prone plaques.^31^ Interestingly, chemokine induced macrophage migration was significantly inhibited by both ERK1 deficiency and STAT1 S727A modification (Figure 2). These findings are surprising given the proposed pro‐atherogenic roles of ERK1 and STAT1 S727 phosphorylation and suggests that the anti‐atherogenic actions of the genetic modifications are potentially mediated via other mechanisms (e.g., proliferation in the case of ERK1 deficiency, chemokine‐driven monocytic migration and recruitment to the activated endothelium in both cases on the basis of gene expression changes of cytokines and cell adhesion molecules observed as shown in Table 1).

Macrophage foam cell formation is a balance between the uptake of modified LDL and the efflux of cholesterol from foam cells.^2^ Our previous study showed that knockdown of STAT1 attenuated both the basal and IFN‐γ induced uptake of modified LDL.^13^ In addition, STAT1 deficiency in mouse macrophages was found to inhibit foam cell formation as determined by lipid staining and the accumulation of cholesteryl esters.^32^ Furthermore, other studies have shown that the IFN‐γ‐mediated suppression of macrophage cholesterol efflux and expression of ABCA1 was mediated via the STAT1 pathway.^33^ Interestingly, *ABCA1* was one of the gene whose expression was significantly decreased in BMDM from STAT1 S727A mice compared to the control (Table 1). Despite this, cholesterol efflux from foam cells, oxLDL uptake, and macropinocytosis were not affected in BMDM from STAT1 S727A mice and phagocytosis was increased (Figure 3). This suggests that either STAT1 S727 phosphorylation has minimal impact on macrophage foam cell formation in vitro or there is potential functional redundancy with tyrosine phosphorylated or unphosphorylated STAT1 present in the cells. However, in vivo, the plaque lipid content was significantly decreased following feeding of HFD for 12 weeks despite no changes in plaque size (Figure 5). This is most likely due to reduced macrophage content of the plaque (Figure 6) potentially because of decreased recruitment of monocytes as a result of dampened inflammation. In support of reduced inflammation, BMDM from STAT1 S727A mice also had significantly decreased gene expression of several pro‐inflammatory cytokines/chemokines/cognate receptors (e.g., *Ccl2*, *Ccr1*, *Ccr2*, *Cxcl1*, *Ifng*, *Il1a*, *Il1b*, *Il1r2*, and *Tnf*; Table 1) compared to control mice. In addition, there were decreased levels of CD3+ T cells in the plaques (Figure 7). Future studies should therefore determine the levels of different T‐cell subtypes (e.g., CD4 T cells, Tregs), different polarized macrophages (e.g., M1 and M2) and other immune cells (e.g., dendritic cells and B cells) in plaques to gain more deeper mechanistic insights especially as inflammation also impacts on plaque stability.

From the plasma cytokine levels, there was a trend toward increase in IL‐5 levels (Table 2) despite reduced expression of the corresponding gene in BMDM of STAT1 S727A mice compared to the control (Table 1). This cytokine has been found to have anti‐atherogenic actions with accelerated atherosclerosis in LDLR^−/−^ mice following bone marrow transplantation from IL‐15 deficient animals.^1^ The plasma levels of IL‐2 were also significantly increased (Table 2). Although IL‐2 is considered as a pro‐inflammatory Th1 cytokine expressed in atherosclerotic plaques, conflicting results have been obtained on its role in the pathogenesis of the disease.^34^ Injection of IL‐2 enhanced atherosclerosis in the Apolipoprotein E deficient (ApoE^−/−^) mouse model whereas the disease was reduced by injection of anti‐IL‐2 antibodies.^1^, ^34^ In contrast, local delivery of IL‐2 to plaques attenuated atherosclerosis development by activating Tregs.^34^ Indeed, a combination therapy with anti‐CD3 antibody and IL‐2/anti‐IL‐2 monoclonal antibody complex aimed at increasing the ratio of Tregs to pathogenic effector T cells inhibited atherosclerosis in the ApoE^−/−^ model system.^35^

Two previous studies have investigated the role of STAT1 in atherosclerosis.^16^, ^32^ Agrawal et al^32^ showed by bone marrow transplantation using ApoE^−/−^ mice that STAT1 deficiency is associated with reduced atherosclerotic lesion formation despite an increase in plasma cholesterol levels and total body weight. In contrast, Lim et al^16^ found no effect of STAT1 deficiency on plasma cholesterol levels, body weight, lesion area or macrophage content following bone marrow transplantation using LDLR^−/−^ mice. However, protective effects were identified on apoptosis in macrophage‐rich lesions and on plaque necrosis.^16^ The exact reasons for the differences between the two studies are currently unclear but could be because of differences in the model systems (ApoE^−/−^ v/s LDLR^−/−^), gender and experimental design such as duration of HFD feeding. Nevertheless, both studies suggest a pro‐atherogenic role of STAT1 (reduced atherosclerotic lesion^32^ or apoptosis/necrosis in macrophage‐rich regions^16^) although they do not inform on whether these are due to phosphorylation of STAT1 on tyrosine 701 or serine 727. However, our study demonstrates that deficiency of STAT1 S727 phosphorylation is associated with reduced plaque lipid, macrophage and CD3+ T‐cell content (Figures 5, 6, 7), thereby indicating the importance of this phosphorylation in several pro‐atherogenic processes. It is also likely that this phosphorylation is potentially responsible for the changes in apoptosis seen in macrophage‐rich areas in the study of Lim et al^16^ given that BMDM from STAT1 S727A mice have decreased expression of several key genes implicated in apoptosis compared to control mice (Table 1). Future studies should therefore investigate the role STAT1 S727 phosphorylation (and ERK1/2 deficiency) on both apoptosis and efferocytosis together with the key signaling pathways involved in these processes.

Gender‐specific actions are relatively common in atherogenesis.^36^ Male mice were used in this study because previous research has shown that deficiency of the cytokine IFN‐γ, which activates ERK1/2 and STAT1, demonstrates gender specific effects on atherosclerosis in ApoE^−/−^ mice.^37^ However, previous bone marrow transplantation studies investigating STAT1 actions has either involved male ApoE^−/−^ mice or female LDLR^−/−^ mice.^16^, ^32^ In the light of such findings, future studies should not only investigate the effects of ERK1 deficiency and STAT1 S727A modification in female LDLR^−/−^ mice but also extend them to both genders in ApoE^−/−^ mice.

The role of the ERK signaling pathway in the control of macrophage foam cell formation in vitro has been suggested by several studies. For example, early growth factor‐1 promoted foam cell formation via the ERK1/2 pathway.^38^ Pharmacological inhibition of ERK1/2 also improved oxLDL‐induced lipid accumulation in rat macrophages.^39^ Interestingly, inhibition of ERK1/2 and the activation of liver X receptor (LXR) was found to synergistically induce macrophage *ABCA1* expression and cholesterol efflux.^40^ However, ERK1 deficiency in BMDM had no significant effect on *ABCA1* expression (Table S1) or cholesterol efflux (Figure 3) compared to those from control mice. In addition, there were no significant differences in phagocytosis or macropinocytosis in BMDM from ERK1^−/−^ mice compared to the control and oxLDL uptake was significantly increased (Figure 3). As pharmacological inhibitors impact both ERK1 and 2, functional redundancy between the two isoforms might potentially be responsible for the lack of responses on some parameters such as cholesterol efflux. However, ERK1 deficiency in vivo was associated with significantly reduced plaque lipid content following 24 weeks feeding of HFD (Figure 5). Although no previous studies have analyzed the effect of ERK1 deficiency on atherosclerosis in vivo, pharmacological inhibition of ERK1/2 and activation of LXR was found to synergistically decrease atherosclerotic lesions in ApoE^−/−^ mice.^41^

Most protective effects of STAT1 S727A modification were observed following feeding of HFD for 12 weeks rather than 24 weeks when compared to the controls (Figures 5, 6, 7). This suggests that inhibition of STAT1 S727A phosphorylation is more effective in limiting pro‐atherogenic parameters induced by HFD feeding in early lesions compared to late lesions. It is possible that extensive compensatory mechanisms take place in advanced lesions due to activation of other STAT family members/transcription factors/kinases because of chronic plaque inflammation via the action of multiple cytokines. Indeed, plaque occlusion was increased in both LDLR^−/−^/ERK1^−/−^ and LDLR^−/−^/STAT1 S727A mice following feeding of HFD for 24 weeks compared to control mice (Figure 5). In addition, the weight of the spleen was decreased (Table S3). The exact role of spleen in atherogenesis is not fully understood although increased spleen size has been associated with decreased atherosclerosis and plaque development in LDLR^−/−^ mice^42^ and splenectomy increases atherosclerotic lesions in ApoE^−/−^ mice due to loss of B‐cell‐associated protective immunity.^43^, ^44^ As the impact of changes in spleen weight is likely to be via modulation of immune cells, future studies should investigate the levels of a range of such cells (e.g., monocytes, CD3 cells, etc) in spleen together with peripheral blood, bone marrow, and thymus.

Despite the loss of anti‐atherogenic effects and some detrimental changes detailed above, there were still some beneficial phenotypes following feeding of the mice with HFD for 24 weeks. For example, HFD‐induced cardiac hypertrophy and heart weight were significantly decreased in LDLR^−/−^/STAT1 S727A mice compared to LDLR^−/−^ controls (Figure 4 and Table S3). The molecular mechanisms underlying decreased cardiac hypertrophy in LDLR^−/−^/STAT1 S727A mice and why these were not seen in ERK1 deficient mice remains to be determined and should be investigated in future studies. Both IFN‐γ and STAT1 have been found to protect against pressure overload‐induced cardiac hypertrophy.^45^, ^46^ Future studies should therefore investigate the effect of STAT1 S727A modification on cardiac hypertrophy induced by sustained pressure overload. In addition, as STAT1 has been previously shown to cause loss of cardiac myocytes by promoting apoptosis and also reducing cardioprotective autophagy,^47^ the role of STAT1 S727 phosphorylation in these and other processes that contribute to cardiac hypertrophy needs to be investigated. ERK1/2 are also involved in cardiomyocyte homeostasis and cardiac stress^48^ so it is possible that functional redundancy between ERK1 and ERK2 could potentially be responsible for the neutral effect. Future studies should therefore also investigate this possibility. Another protective action seen following 24‐week HFD feeding was reduced plaque lipid content in LDLR^−/−^/ERK1^−/−^ mice compared to the controls (Figure 5). Interestingly, atheroprotection through SYK inhibition was found not to occur in advanced lesions when local macrophage proliferation dominates lesion progression.^49^ As the proliferation of BMDM was decreased following ERK1 deficiency (Figure 1), this could represent a potential reason for reduced plaque content in LDLR^−/−^/ERK1 deficient mice following feeding of HFD for 24 weeks.

Decreased plaque macrophage content was seen following 12 weeks feeding of HFD in LDLR^−/−^/STAT1 S727A mice compared to the controls (Figure 6). Multiple mechanisms are likely to contribute to such changes, including reduced migration and recruitment of monocytes due to dampened inflammation. Indeed, the expression of genes encoding Ccr2, the receptor for the key chemokine Ccl2/MCP‐1, and the key pro‐atherogenic cytokine IFN‐γ, both of which play critical roles in monocyte migration and recruitment to the plaques,^1^ was decreased in BMDM from STAT1 S727A mice compared to the control (Table 1). In addition, there was decreased expression of five genes that code for key adhesion proteins involved in cell recruitment (*Cdh5*, *Itga2*, *Lama1*, *Sell*, and *Selplg*; Table 1). Furthermore, the chemokine‐driven migration of BMDM was attenuated by deficiency of STAT1 S727 phosphorylation (Figure 2).

The liver plays an important role in the metabolic perturbations seen in atherosclerosis and other metabolic and inflammatory disorders.^50^ Previous studies have shown an important but complex role of ERK1/2 in the regulation of liver metabolism.^51^ For example, increased ERK activity in diet induced obesity has been shown to potentially contribute to increased glycogen content and reduced energy expenditure in obesity.^52^ ERK1 deficiency in leptin deficient mice also conferred partial protection against hepatic steatosis.^53^ STAT1 has also been found to inhibit mitochondrial biogenesis in the liver and contributes to streptozotocin‐induced diabetic liver injury in mice.^54^, ^55^ In the light of such findings, future studies should investigate the effects of ERK1 deficiency and STAT1 S727A modification on liver metabolism and gene expression, and how these changes then impact atherosclerotic plaque parameters.

Most beneficial effects were observed following deficiency of STAT1 S727A phosphorylation than that of ERK1. Thus, deficiency of STAT1 S727 phosphorylation was associated with reduced plaque content of lipids, macrophages, and CD3+ T cells together with a trend toward increase in plasma IL‐5 levels following feeding of HFD for 12 weeks along with a decrease in diet‐induced hypertrophy index and heart weight after 24 weeks of HFD feeding (Figures 4, 5, 6, 7, Table S3). In addition, the expression of more genes implicated in various atherosclerosis‐associated cellular processes was inhibited in BMDM from STAT1 S727A mice compared to ERK1^−/−^ mice (Table 1). This is surprising given that our previous work using pharmacological agents and knockdown approaches showed that ERK1/2 was required for the IFN‐γ‐mediated activation of STAT1 S727 phosphorylation, the expression of key genes implicated in atherosclerosis, and the uptake of modified lipoproteins by human macrophages.^13^ On this basis, we would have expected similar effects of ERK1 deficiency and STAT1 S727 phosphorylation on atherosclerosis development in vivo. Functional redundancy between ERK1 and ERK2 could potentially have contributed to the less severe impact of ERK1 deficiency. Future studies should therefore seek to understand the effect of both ERK1 and ERK2 deficiency on atherosclerosis development and STAT1 S727‐mediated changes in cellular processes. However, as ERK2 embryos die *in utero*,^18^ conditional knockouts, bone marrow transplantation, or pharmacological inhibition will be required. In addition, further follow‐up studies should extend gene expression changes determined by Atherosclerosis RT^2^ Profiler PCR Arrays to RNA sequencing of BMDM of mouse models used but also others (e.g., global STAT1 deficiency; knock‐in STAT1 mice in which the JAKs‐mediated phosphorylation of tyrosine 701 has been inhibited, ERK2 deficiency and both ERK1/2 deficiency). These will provide comprehensive and more informed comparisons of pathways affected by the various genetic modifications.

Overall, therefore, the studies here suggest that modulation of STAT1 S727 phosphorylation represents a more promising therapeutic option in limiting atherogenesis than ERK1. STAT1 signaling goes beyond IFN‐γ actions; for example, blockade of the cytokine tumor necrosis factor‐like weak inducer of apoptosis also reduced atherosclerotic lesion size and progression via inhibition of STAT1 signaling in diabetic mice.^56^ In addition, transcriptomic analysis of oxLDL stimulated endothelial cells revealed upregulation of STAT1 pathway.^57^ The key role of STAT1 signaling was also suggested by data mining of atherosclerotic plaque transcriptomes^15^ or by differential gene expression analysis of macrophages in atherosclerotic plaques informed by pathways identified via genome‐wide association studies.^14^ Suppressor of cytokine signaling‐1‐derived peptides, which inhibit both STAT‐1 and ‐3 activation, improved inflammation and atherosclerosis in diabetic mice^58^ so this represents one possible therapeutic avenue. However, inactivation of STAT1 by such approaches may make individuals more prone to infection because of drastic effects on the inflammatory response, as has been shown recently in the case of inhibition of IL‐1β in The Canakinumab Anti‐inflammatory Thrombosis Outcome Study.^59^ It might therefore be better to inhibit only STAT1 S727 phosphorylation. This could be achieved by inhibition of key upstream kinases such as ERK1 although this could also have other detrimental effects given the multiple roles of such enzymes, including in the control of inflammation.^17^ A major future challenge is to identify small molecule inhibitors of STAT1 S727 phosphorylation. Interestingly adenosine has been found to inhibit this phosphorylation in response to IFN‐γ leading to decreased macrophage activation.^60^ In addition, our studies have shown that an omega‐6 fatty acid, dihomo‐γ‐linolenic acid (DGLA), which attenuates several atherosclerosis‐associated cellular processes in vitro, inhibits IFN‐γ‐induced STAT1 S727 phosphorylation.^21^ DGLA also attenuates atherosclerosis in ApoE^−/−^ mice although the role of STAT1 S727 phosphorylation has not been investigated.^61^ Thus, the beneficial effects of some nutraceuticals could be, at least in part, via inhibition of STAT1 S727 phosphorylation. In this context, future studies should investigate the action of agents such as DGLA on atherosclerosis‐associated processes on cells from the models used here in vitro and on disease development in vivo.

In conclusion, we show here that deficiency of ERK1 and STAT1 S727 phosphorylation attenuates atherosclerosis‐associated macrophage gene expression together with some parameters of disease development in LDLR^−/−^ mice. As exogenous IFN‐γ has been found to enhance atherosclerosis in ApoE^−/−^ mice fed a normal diet,^1^ follow‐up studies should investigate the impact of ERK1 deficiency and STAT1 serine S727 phosphorylation on disease development following injection of the cytokine in mice. More pronounced effects were seen with deficiency of STAT1 S727 phosphorylation, thereby implicating this as a promising therapeutic target. Future studies should also seek to identify specific inhibitors of this phosphorylation event and evaluate its efficacy in attenuating atherosclerosis and cardiovascular disease initially in pre‐clinical model systems and, if effective, in clinical trials.

## DISCLOSURES

None.

## AUTHOR CONTRIBUTIONS

Wijdan Al‐Ahmadi, Thomas S. Webberley, Thomas Decker, Timothy R. Hughes, and Dipak P. Ramji were responsible for the design of the experiments. Experiments were performed by Wijdan Al‐Ahmadi, Thomas S. Webberley, Alex Joseph, Ffion Harris, Reem Alotibi, Jessica O. Williams and Alaa Alahmadi, and data analysis was carried out by Wijdan Al‐Ahmadi, Thomas S. Webberley, Alex Joseph, Ffion Harris, Yee‐Hung Chan, Reem Alotibi, Jessica O. Williams, and Alaa Alahmadi. Wijdan Al‐Ahmadi, Jessica O. Williams, Yee‐Hung Chan, Reem Alotibi, and Alaa Alahmadi prepared the figures and Wijdan Al‐Ahmadi and Dipak P. Ramji wrote the manuscript. All the authors contributed to the review of the manuscript.

## Supporting information

Supplementary Material
