# Supplementary material for: Pro‐atherogenic actions of signal transducer and activator of transcription 1 serine 727 phosphorylation in LDL receptor deficient mice via modulation of plaque inflammation
Source: FASEB J. 2021 Sep 27;35(10):e21892. doi: 10.1096/fj.202100571RR (PMC9549671; doi:10.1096/fj.202100571RR)
Supplement: Supplementary file 1 — Supplementary Material [file FSB2-35-e21892-s001.pdf]

Supplementary Data

Pro-atherogenic actions of signal transducer and activator of transcription 1 serine 727 phosphorylation in LDL receptor deficient mice via modulation of plaque inflammation

Wijdan Al-Ahmadi[1*], Thomas S. Webberley[1*], Alex Joseph[1], Ffion Harris[1], Yee-Hung Chan[1], Reem Alotibi[1], Jessica O Williams[1], Alaa Alahmadi[1], Thomas Decker[2], Timothy R. Hughes[3] and Dipak P. Ramji[1#]

[1]: Cardiff School of Biosciences, Cardiff University, Sir Martin Evans Building, Museum Avenue, Cardiff CF10 3AX, UK.
[2]: Department of Microbiology and Immunology, Max F. Perutz Laboratories, University of Vienna, Dr. Bohr-Gasse 9, 1030 Vienna, Austria.
[3]. Systems Immunity Research Institute, School of Medicine, Cardiff University, Cardiff CF14 4XN, UK.

*, Joint first authors
[#], To whom correspondence should be addressed: Professor Dipak P. Ramji, Cardiff School of Biosciences, Cardiff University, Sir Martin Evans Building, Museum Avenue, Cardiff CF10 3AX, UK.

Supplementary Table 1. The expression of key atherosclerosis-associated genes in BMDM from ERK1$^{-/-}$ mice compared to those from control C57BL/6J mice

			Control	ERK1 $^{-/-}$		
**#**	**Gene**	**N**	**Mean ± SEM**	**Mean ± SEM**	**% change**	***p* value**
**1**	*Abca1*	6	1.000 ± 0.024	0.890 ± 0.277	11 (↓)	0.902
**2**	*Ace*	5	1.000 ± 0.000	0.589 ± 0.144	41 (↓)	0.044
**3**	*Apoa1*	6	1.000 ± 0.001	0.383 ± 0.125	62 (↓)	0.003
**4**	*Apob*	6	1.000 ± 0.004	0.621 ± 0.185	38 (↓)	0.094
**5**	*Apoe*	6	1.000 ± 0.091	3.209 ± 1.231	221 (↑)	0.116
**6**	*Bax*	6	1.000 ± 0.019	0.892 ± 0.251	11 (↓)	0.990
**7**	*Bcl2*	4	1.000 ± 0.001	0.603 ± 0.168	40 (↓)	0.086
**8**	*Bcl2a1a*	6	1.000 ± 0.001	2.274 ± 0.807	127 (↑)	0.164
**9**	*Bcl2l1*	6	1.000 ± 0.053	0.565 ± 0.122	44 (↓)	0.174
**10**	*Bid*	6	1.000 ± 0.013	0.739 ± 0.178	26 (↓)	0.412
**11**	*Birc3*	6	1.000 ± 0.004	0.880 ± 0.265	12 (↓)	0.634
**12**	*Ccl2*	6	1.000 ± 0.001	1.863 ± 0.524	86 (↑)	0.139
**13**	*Ccl5*	6	1.000 ± 0.000	2.093 ± 1.037	109 (↑)	0.350
**14**	*Ccr1*	5	1.000 ± 0.009	0.682 ± 0.280	32 (↓)	0.463
**15**	*Ccr2*	5	1.000 ± 0.000	0.301 ± 0.079	70 (↓)	0.087
**16**	*Cd44*	6	1.000 ± 0.087	0.677 ± 0.167	32 (↓)	0.443
**17**	*Cdh5*	6	1.000 ± 0.000	0.434 ± 0.111	57 (↓)	0.002
**18**	*Cflar*	6	1.000 ± 0.021	0.919 ± 0.281	8 (↓)	0.937
**19**	*Col3a1*	4	1.000 ± 0.002	4.343 ± 1.536	334 (↑)	0.095
**20**	*Csf2*	3	1.000 ± 0.001	0.429 ± 0.088	57 (↓)	<0.001
**21**	*Ctgf*	6	1.000 ± 0.001	5.627 ± 2.322	463 (↑)	0.104
**22**	*Cxcl1*	4	UD	UD	N/A	N/A
**23**	*Eln*	4	1.000 ± 0.003	0.195 ± 0.035	81 (↓)	<0.001
**24**	*Eng*	6	1.000 ± 0.008	0.751 ± 0.174	25 (↓)	0.395
**25**	*Fabp3*	6	1.000 ± 0.001	2.210 ± 0.814	121 (↑)	0.172
**26**	*Fas*	6	1.000 ± 0.001	1.270 ± 0.382	27 (↑)	0.509
**27**	*Fga*	6	1.000 ± 0.001	0.679 ± 0.184	32 (↓)	0.157
**28**	*Fgb*	6	1.000 ± 0.002	0.384 ± 0.100	62 (↓)	0.001
**29**	*Fgf2*	6	1.000 ± 0.004	0.825 ± 0.181	18 (↓)	0.339
**30**	*Fn1*	6	1.000 ± 0.001	1.399 ± 0.630	40 (↑)	0.590
**31**	*Hbegf*	6	1.000 ± 0.001	0.563 ± 0.159	44 (↓)	0.043
**32**	*Icam1*	6	1.000 ± 0.010	1.520 ± 0.520	52 (↑)	0.367
**33**	*Ifng*	6	1.000 ± 0.004	0.544 ± 0.165	46 (↓)	0.039
**34**	*Il1a*	6	1.000 ± 0.000	2.780 ± 1.104	178 (↑)	0.174
**35**	*Il1b*	6	1.000 ± 0.001	1.169 ± 0.638	17 (↑)	0.845
**36**	*Il1r1*	6	1.000 ± 0.004	0.964 ± 0.308	4 (↓)	0.906
**37**	*Il1r2*	5	1.000 ± 0.003	0.647 ± 0.372	35 (↓)	0.354
**38**	*Il2*	6	1.000 ± 0.003	0.789 ± 0.287	21 (↓)	0.470
**39**	*Il3*	3	1.000 ± 0.001	0.233 ± 0.052	77 (↓)	<0.001
**40**	*Il4*	4	1.000 ± 0.000	0.458 ± 0.132	54 (↓)	0.007
**41**	*Il5*	3	1.000 ± 0.000	0.243 ± 0.055	76 (↓)	<0.001

**42**	*Itga2*	5	1.000 ± 0.000	0.395 ± 0.089	61 (↓)	<0.001
**43**	*Itga5*	4	1.000 ± 0.029	0.714 ± 0.174	29 (↓)	0.404
**44**	*Itgax*	4	1.000 ± 0.071	0.560 ± 0.182	44 (↓)	0.269
**45**	*Itgb2*	6	1.000 ± 0.029	1.296 ± 0.386	30 (↑)	0.488
**46**	*Kdr*	6	1.000 ± 0.003	1.973 ± 0.688	97 (↑)	0.188
**47**	*Klf2*	6	UD	UD	N/A	N/A
**48**	*Lama1*	6	1.000 ± 0.003	0.448 ± 0.145	55 (↓)	0.008
**49**	*Ldlr*	4	1.000 ± 0.024	0.771 ± 0.170	23 (↓)	0.579
**50**	*Lif*	4	1.000 ± 0.001	1.000 ± 0.310	0	0.964
**51**	*Lpl*	6	1.000 ± 0.132	1.423 ± 0.304	42 (↑)	0.396
**52**	*Lypla1*	6	1.000 ± 0.012	0.823 ± 0.202	18 (↓)	0.720
**53**	*Mmp1a*	6	1.000 ± 0.138	1.739 ± 0.372	74 (↑)	0.241
**54**	*Mmp3*	4	1.000 ± 0.001	3.700 ± 1.453	270 (↑)	0.138
**55**	*Msr1*	4	1.000 ± 0.102	0.659 ± 0.160	34 (↓)	0.461
**56**	*Nfkb1*	6	1.000 ± 0.009	0.636 ± 0.207	36 (↓)	0.205
**57**	*Npy*	4	1.000 ± 0.010	5.065 ± 1.607	407 (↑)	0.050
**58**	*Nr1h3*	5	1.000 ± 0.014	0.863 ± 0.287	14 (↓)	0.799
**59**	*Pdgfa*	5	1.000 ± 0.003	1.110 ± 0.313	11 (↑)	0.555
**60**	*Pdgfb*	5	1.000 ± 0.023	0.613 ± 0.132	39 (↓)	0.131
**61**	*Pdgfrb*	6	1.000 ± 0.002	0.864 ± 0.319	14 (↓)	0.657
**62**	*Plin2*	6	1.000 ± 0.090	1.610 ± 0.466	61 (↑)	0.274
**63**	*Ppara*	6	1.000 ± 0.002	0.310 ± 0.107	69 (↓)	<0.001
**64**	*Ppard*	5	1.000 ± 0.001	0.903 ± 0.267	10 (↓)	0.897
**65**	*Pparg*	6	1.000 ± 0.037	0.694 ± 0.198	31 (↓)	0.445
**66**	*Ptgs1*	6	1.000 ± 0.002	0.961 ± 0.222	4 (↓)	0.976
**67**	*Rxra*	6	1.000 ± 0.009	0.763 ± 0.247	24 (↓)	0.529
**68**	*Sele*	4	1.000 ± 0.000	0.399 ± 0.161	60 (↓)	0.009
**69**	*Sell*	5	1.000 ± 0.003	0.836 ± 0.020	16 (↓)	0.071
**70**	*Selp*	5	1.000 ± 0.000	0.854 ± 0.081	15 (↓)	0.122
**71**	*Selpg*	6	1.000 ± 0.051	0.425 ± 0.092	58 (↓)	0.019
**72**	*Serpinb2*	4	1.000 ± 0.001	1.812 ± 0.755	81 (↑)	0.306
**73**	*Serpine1*	6	1.000 ± 0.006	2.397 ± 1.011	140 (↑)	0.226
**74**	*Sod1*	6	1.000 ± 0.041	0.673 ± 0.169	33 (↓)	0.339
**75**	*Spp1*	6	1.000 ± 0.128	4.617 ± 1.514	362 (↑)	0.062
**76**	*Tgfb1*	3	1.000 ± 0.132	0.863 ± 0.212	14 (↓)	0.706
**77**	*Tgfb2*	5	1.000 ± 0.003	0.966 ± 0.260	3 (↓)	0.851
**78**	*Thbs4*	3	1.000 ± 0.002	0.647 ± 0.332	35 (↓)	0.388
**79**	*Tnc*	6	1.000 ± 0.004	2.011 ± 0.894	101 (↑)	0.321
**80**	*Tnf*	5	1.000 ± 0.012	1.183 ± 0.329	18 (↑)	0.562
**81**	*Tnfaip3*	4	1.000 ± 0.002	1.765 ± 0.429	77 (↑)	0.116
**82**	*Vcam1*	6	1.000 ± 0.001	2.798 ± 1.152	180 (↑)	0.197
**83**	*Vegfa*	6	1.000 ± 0.007	0.436 ± 0.122	56 (↓)	0.004
**84**	*Vwf*	3	1.000 ±0.006	1.372 ± 0.456	37 (↑)	0.483
	Actb	6	1.000 ± 0.018	1.068 ± 0.012	7 (↑)	0.596
	B2m	6	1.000 ± 0.048	1.084 ± 0.029	8 (↑)	0.640
	Gapdh	6	1.000 ± 0.041	1.087 ± 0.009	9 (↑)	0.676

Gusb	6	1.000 ± 0.019	1.053 ± 0.095	5 (↑)	0.515
Hsp90ab1	6	1.000 ± 0.020	1.061 ± 0.010	6 (↑)	0.551

Gene expression was normalized to the three most stable house-keeping genes; Actb, Gusb and Hsp90ab1 (SEM of 0.01-0.02 and approximately 5-7% change in expression). The value in BMDM from control mice has been arbitrarily assigned as 1 for each gene. Statistical analysis was performed using an unpaired Student's t-test and the *p* values are shown. The percentage change in expression of each gene is also shown with ↑ indicating induction and ↓ showing reduction in expression. UD, undetectable. *Abbreviations*: *Abca1*, ATP-binding cassette, subfamily A (ABC1), member 1; *Ace*, angiotensin I converting enzyme (peptidyl-dipeptidase A) 1; *ACTB*, β-actin; *Apoa1*, apolipoprotein A-I; *Apob*, apolipoprotein B; *Apoe*, apolipoprotein E; *B2M*, β-2-microglobulin; *Bax*, BCL2-associated X protein; *Bcl2*, B cell leukemia/lymphoma 1; *Bcl2a1a* (Bfl-1, A1), B cell leukemia/lymphoma 2 related protein A1a; *Bcl2l1* (Bcl-XL), BCL2-like 1; *Bid*, BH3 interacting domain death agonist; *Birc3* (cIAP1, cIAP2), buculoviral IAP repeat-containing 3; *Ccl2* (MCP-1), chemokine (C-C motif) ligand 2; *Ccl5* (RANTES), chemokine (C-C motif) ligand 5; *Ccr1*, chemokine (C-C motif) receptor 1; *Ccr2*, chemokine (C-C motif) receptor 2; *Cd44*, CD44 antigen; *Cdh5*, cadherin 5; *Cflar* (Casper), CASP8 and FADD-like apoptosis regulator; *Col3a1*, collagen, type III, α1; *Csf2* (GMCSF), colony stimulating factor 2 (granulocyte-macrophage); *Ctgf*, connective tissue growth factor; *Cxcl1* (Gro1), chemokine (C-X-C motif) ligand 1; *Eln*, elastin; Eng1 (Evi-1), endoglin; *Fabp3*, fatty acid binding protein 3, muscle and heart; *Fas* (TNFRSF6), TNF receptor superfamily member 6; *Fga*, fibrinogen α chain; *Fgb*, fibrinogen β chain; *Fgf2* (bFGF), fibroblast growth factor 2; *Fn1*, fibronectin 1; *GAPDH*, glyceraldehyde 3-phosphate dehydrogenase; *GUSB*, β-glucoronidase; Hbegf (Dtr), heparin-binding EGF-like growth factor; *HSP90AB1*, heat shock protein 90β; *Icam1*, intercellular adhesion molecule 1; *Ifng*, interferon-γ; *Il1a*, interleukin-1α; *Il1b*, interleukin-1β; *Il1r1*, interleukin 1 receptor, type I; *Il1r2*, interleukin 1 receptor, type II; *Il2*, interleukin-2; *Il3*, interleukin-3; *Il4*, interleukin-4; *Il5*, interleukin-5; *Itga2*, integrin α2; *Itga5*, integrin α 5 (fibronectin receptor α); *Itgax*, integrin α X; *Itgb2*, integrin β2; *Kdr* (VEGFR2), kinase insert domain protein receptor; *Klf2*, kruppel-like factor 2 (lung); *Lama1*, laminin α1; *Ldlr*, low density lipoprotein receptor; *Lif*, leukemia inhibitory factor; *Lpl*, lipoprotein lipase; *Lypla1*, lysophospholipase 1; *Mmp1a*, matrix metallopeptidase 1a (interstitial collagenase); *Mmp3*, matrix metallopeptidase 3; *Msr1*, macrophage scavenger receptor 1; *Nfkb1*, nuclear factor of kappa light polypeptide gene enhancer in B cells 1, p105; *Npy*, neuropeptide Y; *Nr1h3*, nuclear receptor subfamily 1, h group H, member 3; *Pdgfa*, platelet derived growth factor α; *Pdgfb*, platelet derived growth factor, B polypeptide; *Pdgfrb*, platelet derived growth factor receptor, beta polypeptide; *Plin2*, perlipin 2; *Ppara*, peroxisome proliferator activated receptor α; *Ppard*, peroxisome proliferator activated receptor δ; *Pparg*, peroxisome proliferator activated receptor γ; *Ptgs1* (COX1), prostaglandin-endoperoxide synthase 1; *Rxra*, retinoid X receptor α; *Sele*, selectin, endothelial cells; *Sell* (LECAM-1), selectin, lymphocyte; *Selp*, selectin, platelet; *Selpg* (P-Selectin), selectin, platelet (p-selectin) ligand; *Serpinb2* (PAI-2), serine (or cysteine) peptidase inhibitor, clade B, member 2; *Serpine1* (PAI-1), serine (or cysteine) peptidase inhibitor, clade B, member 1; *Sod1*, superoxide dismutase 1; *Spp1*, secreted phosphoprotein 1; *Tgfb1*, transforming growth factor-β1; *Tgfb2*, transforming growth factor-β2; *Thbs4*, thrombospondin 4; *Tnc*, tenascin C; *Tnf*, tumor necrosis factor; *Tnfaip3*, tumor necrosis factor, alpha-induced protein 3; *Vcam1*, vascular cell adhesion molecule 1; *Vegfa*, vascular endothelial growth factor A; *Vwf*, Von Willebrand factor

Supplementary Table 2. The expression of key atherosclerosis-associated genes in BMDM from STAT1 S727A mice compared to those from control C57BL/6J mice

			Control	STAT1 S727A		
**#**	**Gene**	**N**	**Mean ± SEM**	**Mean ± SEM**	**% change**	***p* value**
**1**	*Abca1*	6	1.000 ± 0.367	0.449 ± 0.167	55 (↓)	0.021
**2**	*Ace*	4	1.000 ± 0.002	0.003 ± 0.002	100 (↓)	<0.001
**3**	*Apoa1*	4	1.000 ± 0.039	0.057 ± 0.049	94 (↓)	<0.001
**4**	*Apob*	5	1.000 ± 0.046	0.406 ± 0.301	59 (↓)	0.120
**5**	*Apoe*	4	1.000 ± 0.008	0.867 ± 0.682	13 (↓)	0.858
**6**	*Bax*	6	1.000 ± 0.265	0.537 ± 0.224	46 (↓)	0.093
**7**	*Bcl2*	3	1.000 ± 0.025	0.002 ± 0.002	100 (↓)	<0.001
**8**	*Bcl2a1a*	3	1.000 ± 0.254	0.010 ± 0.008	99 (↓)	<0.001
**9**	*Bcl2l1*	5	1.000 ± 0.195	0.302 ± 0.127	70 (↓)	0.006
**10**	*Bid*	5	1.000 ± 1.290	0.505 ± 0.301	50 (↓)	0.175
**11**	*Birc3*	3	1.000 ± 0.097	0.311 ± 0.310	69 (↓)	0.112
**12**	*Ccl2*	4	1.000 ± 0.226	0.086 ± 0.081	91 (↓)	0.002
**13**	*Ccl5*	5	1.000 ± 0.072	0.864 ± 0.690	14 (↓)	0.853
**14**	*Ccr1*	4	1.000 ± 0.042	0.132 ± 0.132	87 (↓)	0.007
**15**	*Ccr2*	4	1.000 ± 0.000	0.014 ± 0.011	99 (↓)	<0.001
**16**	*Cd44*	6	1.000 ± 0.739	0.423 ± 0.250	58 (↓)	0.069
**17**	*Cdh5*	3	1.000 ± 0.021	0.025 ± 0.022	98 (↓)	0.014
**18**	*Cflar*	6	1.000 ± 0.029	0.971 ± 0.672	3 (↓)	0.967
**19**	*Col3a1*	1	1.000	UD	N/A	N/A
**20**	*Csf2*	5	1.000 ± 0.018	0.005 ± 0.004	100 (↓)	<0.001
**21**	*Ctgf*	6	1.000 ± 0.119	0.100 ± 0.053	90 (↓)	<0.001
**22**	*Cxcl1*	3	1.000 ± 0.255	0.006 ± 0.000	99 (↓)	<0.001
**23**	*Eln*	5	1.000 ± 0.047	0.393 ± 0.309	61 (↓)	0.121
**24**	*Eng*	5	1.000 ± 0.077	0.528 ± 0.347	47 (↓)	0.245
**25**	*Fabp3*	5	1.000 ± 0.278	0.709 ± 0.559	29 (↓)	0.629
**26**	*Fas*	5	1.000 ± 0.111	0.192 ± 0.126	81 (↓)	0.003
**27**	*Fga*	5	1.000 ± 0.017	0.137 ± 0.082	86 (↓)	<0.001
**28**	*Fgb*	5	1.000 ± 0.025	0.078 ± 0.075	92 (↓)	0.009
**29**	*Fgf2*	4	1.000 ± 0.046	0.144 ± 0.141	86 (↓)	0.009
**30**	*Fn1*	5	1.000 ± 0.011	0.705 ± 0.480	30 (↓)	0.573
**31**	*Hbegf*	5	1.000 ± 0.062	0.284 ± 0.172	72 (↓)	0.014
**32**	*Icam1*	4	1.000 ± 0.090	0.418 ± 0.292	58 (↓)	0.140
**33**	*Ifng*	4	1.000 ± 0.058	0.095 ± 0.086	91 (↓)	0.002
**34**	*Il1a*	4	1.000 ± 0.148	0.011 ± 0.008	99 (↓)	<0.001
**35**	*Il1b*	5	1.000 ± 0.019	0.206 ± 0.122	79 (↓)	0.003
**36**	*Il1r1*	4	1.000 ± 0.077	0.333 ± 0.227	67 (↓)	0.061
**37**	*Il1r2*	5	1.000 ± 0.087	0.229 ± 0.176	77 (↓)	0.012
**38**	*Il2*	5	1.000 ± 0.016	0.354 ± 0.245	65 (↓)	0.057
**39**	*Il3*	5	1.000 ± 0.040	0.356 ± 0.219	64 (↓)	0.042
**40**	*Il4*	5	1.000 ± 0.039	0.472 ± 0.464	53 (↓)	0.319
**41**	*Il5*	4	1.000 ± 0.454	0.026 ± 0.019	97 (↓)	<0.001

**42**	*Itga2*	5	1.000 ± 0.077	0.318 ± 0.260	68 (↓)	0.059
**43**	*Itga5*	6	1.000 ± 0.244	0.414 ± 0.165	59 (↓)	0.016
**44**	*Itgax*	5	1.000 ± 0.890	0.244 ± 0.140	76 (↓)	0.006
**45**	*Itgb2*	6	1.000 ± 0.108	0.733 ± 0.464	27 (↓)	0.590
**46**	*Kdr*	5	1.000 ± 0.205	0.463 ± 0.353	54 (↓)	0.203
**47**	*Klf2*	6	UD	UD	N/A	N/A
**48**	*Lama1*	4	1.000 ± 0.051	0.182 ± 0.180	82 (↓)	0.020
**49**	*Ldlr*	6	1.000 ± 0.136	0.618 ± 0.312	38 (↓)	0.276
**50**	*Lif*	4	1.000 ± 0.020	0.101 ± 0.095	90 (↓)	0.002
**51**	*Lpl*	6	1.000 ± 0.486	0.510 ± 0.239	49 (↓)	0.095
**52**	*Lypla1*	6	1.000 ± 0.113	0.279 ± 0.133	72 (↓)	0.003
**53**	*Mmp1a*	6	1.000 ± 6.998	0.437 ± 0.196	56 (↓)	0.035
**54**	*Mmp3*	5	1.000 ± 0.044	0.376 ± 0.235	62 (↓)	0.056
**55**	*Msr1*	6	1.000 ± 0.322	1.131 ± 0.452	13 (↑)	0.783
**56**	*Nfkb1*	5	1.000 ± 0.213	0.480 ± 0.204	52 (↓)	0.064
**57**	*Npy*	4	1.000 ± 0.213	0.412 ± 0.339	59 (↓)	0.181
**58**	*Nr1h3*	5	1.000 ± 0.111	0.320 ± 0.179	68 (↓)	0.019
**59**	*Pdgfa*	5	1.000 ± 0.354	0.127 ± 0.090	87 (↓)	0.001
**60**	*Pdgfb*	5	1.000 ± 0.149	0.269 ± 0.133	73 (↓)	0.001
**61**	*Pdgfrb*	6	1.000 ± 0.163	0.199 ± 0.151	80 (↓)	0.006
**62**	*Plin2*	5	1.000 ± 0.535	0.264 ± 0.130	74 (↓)	0.004
**63**	*Ppara*	5	1.000 ± 0.026	0.170 ± 0.144	83 (↓)	0.004
**64**	*Ppard*	5	1.000 ± 0.472	0.107 ± 0.084	89 (↓)	<0.001
**65**	*Pparg*	5	1.000 ± 2.014	0.952 ± 0.768	5 (↓)	0.953
**66**	*Ptgs1*	4	1.000 ± 0.252	0.087 ± 0.065	91 (↓)	<0.001
**67**	*Rxra*	6	1.000 ± 1.853	0.409 ± 0.235	59 (↓)	0.053
**68**	*Sele*	4	1.000 ± 0.038	2.194 ± 2.127	119 (↑)	0.614
**69**	*Sell*	5	1.000 ± 0.001	0.049 ± 0.046	95 (↓)	<0.001
**70**	*Selp*	4	1.000 ± 0.035	0.405 ± 0.266	60 (↓)	0.112
**71**	*Selpg*	6	1.000 ± 6.754	0.291 ± 0.130	71 (↓)	0.003
**72**	*Serpinb2*	5	1.000 ± 0.010	0.707 ± 0.583	29 (↓)	0.642
**73**	*Serpine1*	5	1.000 ± 0.010	0.481 ± 0.339	52 (↓)	0.201
**74**	*Sod1*	6	1.000 ± 0.420	0.522 ± 0.330	48 (↓)	0.207
**75**	*Spp1*	4	1.000 ± 0.023	1.557 ± 0.841	56 (↑)	0.555
**76**	*Tgfb1*	6	1.000 ± 0.025	0.368 ± 0.179	63 (↓)	0.017
**77**	*Tgfb2*	5	1.000 ± 0.157	0.101 ± 0.097	90 (↓)	0.001
**78**	*Thbs4*	5	1.000 ± 0.012	0.005 ± 0.003	100 (↓)	<0.001
**79**	*Tnc*	4	1.000 ± 0.168	0.064 ± 0.062	94 (↓)	<0.001
**80**	*Tnf*	5	1.000 ± 0.156	0.252 ± 0.158	75 (↓)	0.009
**81**	*Tnfaip3*	5	1.000 ± 0.410	0.256 ± 0.256	74 (↓)	0.044
**82**	*Vcam1*	4	1.000 ± 0.133	0.002 ± 0.002	100 (↓)	<0.001
**83**	*Vegfa*	6	1.000 ± 0.003	0.401 ± 0.231	60 (↓)	0.049
**84**	*Vwf*	3	1.000 ±0.036	0.001 ± 0.001	100 (↓)	<0.001
	Actb	6	1.000 ± 0.050	0.991 ± 0.002	1 (↓)	0.487
	B2m	3	1.000 ± 0.036	0.967 ± 0.010	3 (↓)	0.322
	Gapdh	6	1.000 ± 0.028	0.801 ± 0.018	20 (↓)	0.058

Gusb	6	1.000 ± 0.021	0.820 ± 0.009	18 (↓)	0.163
Hsp90ab1	6	1.000 ± 0.027	0.862 ± 0.020	14 (↓)	0.070

Gene expression was normalized to the two most stable house-keeping genes; ACTB and B2M (low SEM and approximately 1-3% change in expression). The value in BMDM from control mice has been arbitrarily assigned as 1 for each gene. Statistical analysis was performed using an unpaired Student's t-test and the *p* values are shown. The percentage change in expression of each gene is also shown with ↑ indicating induction and ↓ showing reduction in expression. UD, undetectable. *Abbreviations*: *Abca1*, ATP-binding cassette, subfamily A (ABC1), member 1; *Ace*, angiotensin I converting enzyme (peptidyl-dipeptidase A) 1; *ACTB*, β-actin; *Apoa1*, apolipoprotein A-I; *Apob*, apolipoprotein B; *Apoe*, apolipoprotein E; *B2M*, β-2-microglobulin; *Bax*, BCL2-associated X protein; *Bcl2*, B cell leukemia/lymphoma 1; *Bcl2a1a* (Bfl-1, A1), B cell leukemia/lymphoma 2 related protein A1a; *Bcl2l1* (Bcl-XL), BCL2-like 1; *Bid*, BH3 interacting domain death agonist; *Birc3* (cIAP1, cIAP2), buculoviral IAP repeat-containing 3; *Ccl2* (MCP-1), chemokine (C-C motif) ligand 2; *Ccl5* (RANTES), chemokine (C-C motif) ligand 5; *Ccr1*, chemokine (C-C motif) receptor 1; *Ccr2*, chemokine (C-C motif) receptor 2; *Cd44*, CD44 antigen; *Cdh5*, cadherin 5; *Cflar* (Casper), CASP8 and FADD-like apoptosis regulator; *Col3a1*, collagen, type III, α1; *Csf2* (GMCSF), colony stimulating factor 2 (granulocyte-macrophage); *Ctgf*, connective tissue growth factor; *Cxcl1* (Gro1), chemokine (C-X-C motif) ligand 1; *Eln*, elastin; Eng1 (Evi-1), endoglin; *Fabp3*, fatty acid binding protein 3, muscle and heart; *Fas* (TNFRSF6), TNF receptor superfamily member 6; *Fga*, fibrinogen α chain; *Fgb*, fibrinogen β chain; *Fgf2* (bFGF), fibroblast growth factor 2; *Fn1*, fibronectin 1; *GAPDH*, glyceraldehyde 3-phosphate dehydrogenase; *GUSB*, β-glucoronidase; Hbegf (Dtr), heparin-binding EGF-like growth factor; *HSP90AB1*, heat shock protein 90β; *Icam1*, intercellular adhesion molecule 1; *Ifng*, interferon-γ; *Il1a*, interleukin-1α; *Il1b*, interleukin-1β; *Il1r1*, interleukin 1 receptor, type I; *Il1r2*, interleukin 1 receptor, type II; *Il2*, interleukin-2; *Il3*, interleukin-3; *Il4*, interleukin-4; *Il5*, interleukin-5; *Itga2*, integrin α2; *Itga5*, integrin α 5 (fibronectin receptor α); *Itgax*, integrin α X; *Itgb2*, integrin β2; *Kdr* (VEGFR2), kinase insert domain protein receptor; *Klf2*, kruppel-like factor 2 (lung); *Lama1*, laminin α1; *Ldlr*, low density lipoprotein receptor; *Lif*, leukemia inhibitory factor; *Lpl*, lipoprotein lipase; *Lypla1*, lysophospholipase 1; *Mmp1a*, matrix metallopeptidase 1a (interstitial collagenase); *Mmp3*, matrix metallopeptidase 3; *Msr1*, macrophage scavenger receptor 1; *Nfkb1*, nuclear factor of kappa light polypeptide gene enhancer in B cells 1, p105; *Npy*, neuropeptide Y; *Nr1h3*, nuclear receptor subfamily 1, h group H, member 3; *Pdgfa*, platelet derived growth factor α; *Pdgfb*, platelet derived growth factor, B polypeptide; *Pdgfrb*, platelet derived growth factor receptor, beta polypeptide; *Plin2*, perlipin 2; *Ppara*, peroxisome proliferator activated receptor α; *Ppard*, peroxisome proliferator activated receptor δ; *Pparg*, peroxisome proliferator activated receptor γ; *Ptgs1* (COX1), prostaglandin-endoperoxide synthase 1; *Rxra*, retinoid X receptor α; *Sele*, selectin, endothelial cells; *Sell* (LECAM-1), selectin, lymphocyte; *Selp*, selectin, platelet; *Selpg* (P-Selectin), selectin, platelet (p-selectin) ligand; *Serpinb2* (PAI-2), serine (or cysteine) peptidase inhibitor, clade B, member 2; *Serpine1* (PAI-1), serine (or cysteine) peptidase inhibitor, clade B, member 1; *Sod1*, superoxide dismutase 1; *Spp1*, secreted phosphoprotein 1; *Tgfb1*, transforming growth factor-β1; *Tgfb2*, transforming growth factor-β2; *Thbs4*, thrombospondin 4; *Tnc*, tenascin C; *Tnf*, tumor necrosis factor; *Tnfaip3*, tumor necrosis factor, alpha-induced protein 3; *Vcam1*, vascular cell adhesion molecule 1; *Vegfa*, vascular endothelial growth factor A; *Vwf*, Von Willebrand factor

Supplementary Table 3. Plasma lipid profile and organ weights in LDLR$^{-/-}$, LDLR$^{-/-}$/ERK1$^{-/-}$ and LDLR$^{-/-}$/STAT1 S727A mice following feeding of HFD

Parameter	LDLR$^{-/-}$	N	LDLR$^{-/-}$ /ERK1$^{-/-}$	N	Change	LDLR$^{-/-}$ /STAT1 S727A	N	Change
**Heart Weight (12 weeks)**	0.52±0.02	15	0.48±0.02	11	NS	0.47±0.02	15	NS
**Heart Weight (24 weeks)**	0.56±0.04	13	0.49±0.03	15	NS	0.36±0.01	15	↓ (p<0.001)
**Liver Weight (12 weeks)**	4.35±0.09	15	4.25±0.13	11	NS	4.30±0.11	12	NS
**Liver Weight (24 weeks)**	4.41±0.11	13	4.13±0.17	15	NS	4.83±0.28	15	NS
**Spleen Weight (12 Weeks)**	0.34±0.03	15	0.29±0.02	15	NS	0.24±0.01	15	NS
**Spleen Weight (24 Weeks)**	0.40±0.05	13	0.30±0.02	15	↓ (*p*= 0.032)	0.24±0.01	15	↓(p<0.001)
**TC (12 Weeks)**	1122.40±41.00	15	983.60±86.00	11	NS	1082.00±73.87	12	NS
**TC (24 Weeks)**	934.80±61.00	13	937.30±35.80	15	NS	881.30±44.30	15	NS
**LDL/VLDL (12 Weeks)**	83.19±3.76	15	90.11±4.75	11	NS	84.76±4.87	12	NS
**LDL/VLDL (24 Weeks)**	77.72±8.05	13	103.20±6.10	15	↑ (p=0.011)	90.49±4.07	15	NS
**HDL (12 Weeks)**	69.50±2.70	15	71.58±4.20	11	NS	76.50±4.60	12	NS
**HDL (24 Weeks)**	73.86±2.26	13	71.38±4.34	15	NS	73.03±2.37	11	NS
**TG (12 Weeks)**	3.84±0.38	15	7.27±1.04	10	↑ (*p*= 0.001)	6.06±0.50	9	↑ (*p*=0.039)
**TG (24 Weeks)**	3.31±0.49	6	6.40±0.65	14	↑ (*p*=0.007)	5.08±0.52	10	NS

Significant or trend of increase (↑) or decrease (↓) are shown with *p* values in parenthesis. NS, not significant; N, numbers of animals. The organ weights are expressed as percentage of body weight and lipids are in mg/dl.

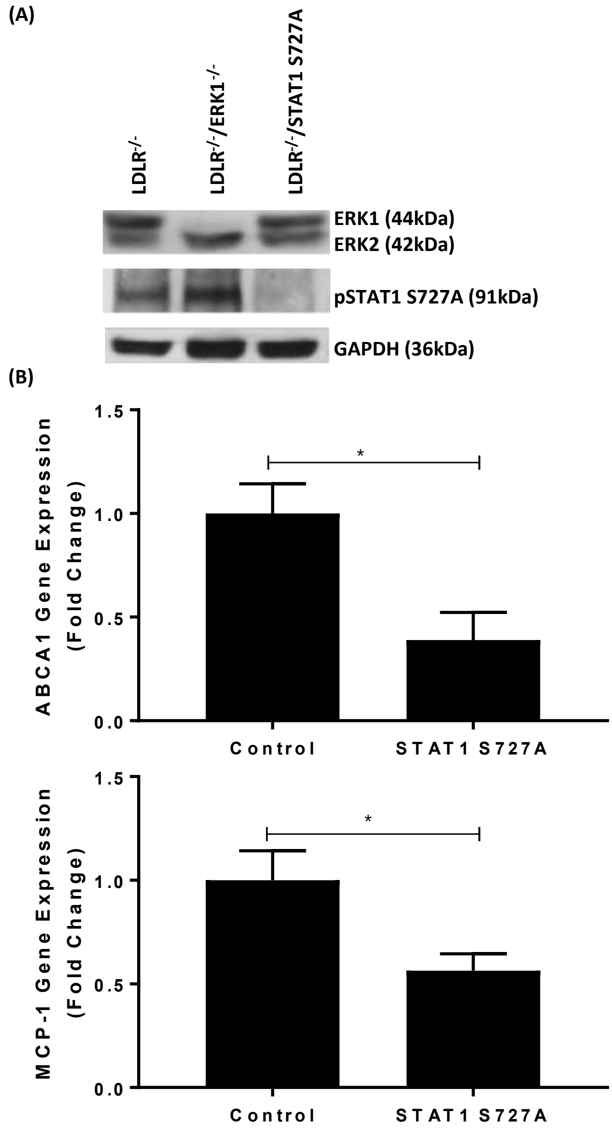

Supplementary Figure 1. Confirmation of the genetic modifications in ERK1$^{-/-}$ and STAT1 S727A mice and the effect of STAT1 S727A modification on the expression of *ABCA1* and *MCP-1* (*CCL2*) genes in BMDM

(A) Total protein lysates were prepared from the livers of C57BL/6J mice (Control), ERK1$^{-/-}$ mice or STAT1 S727A mice and subjected to SDS PAGE and Western blot analysis. The blots were then probed with antibodies against ERK1/2, phospho-STAT1 serine 727 or GAPDH as housekeeping protein. The image shows the immunoreactive proteins with the corresponding molecular weights shown on the right side in parenthesis. **(B)** BMDM were isolated from C57BL/6J (Control) or STAT1 S727A mice and cultured for 24 h. Total RNA was isolated, reverse transcribed to cDNA and used for RT-qPCR with primers against *ABCA1*, *MCP-1* or the *β-actin* control. The mRNA levels were calculated using the comparative Ct method and normalized to β-actin with values from BMDM of control mice given an arbitrary value of 1. The data (mean ± SEM) are from four (*MCP-1*) or six (*ABCA1*) independent experiments. Statistical analysis was performed using an unpaired Student's t test (*, $p \leq 0.05$).

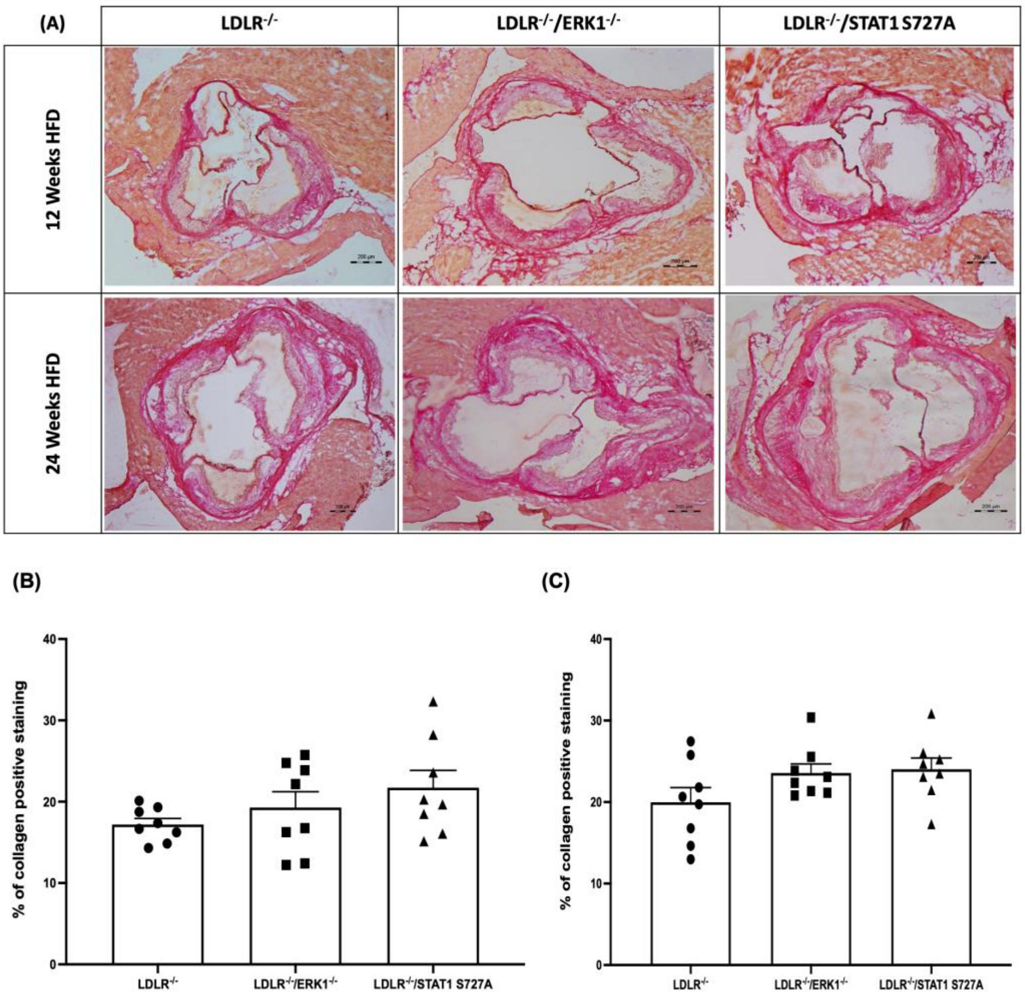

Supplementary Figure 2. The effect of ERK1 deficiency and STAT1 S727A modification on plaque collagen content

LDLR$^{-/-}$, LDLR$^{-/-}$/ERK1$^{-/-}$ or LDLR$^{-/-}$/STAT1 S727A mice were fed HFD for 12- or 24-weeks. Sections of the aortic root were stained for collagen content using Van Gieson's stain. Representative images are shown in panel **A** (5 x magnification and scale bar of 200 μm). The graphs show mean ± SEM of % collagen content within the plaque for LDLR$^{-/-}$, LDLR$^{-/-}$/ERK1$^{-/-}$ or LDLR$^{-/-}$/STAT1 S727A mice fed HFD for 12 weeks **(B)** or 24 weeks **(C)**; n=8 in all cases]. Statistical analysis was performed using a one-way ANOVA with Tukey's post hoc test.
